# Prevalence of suicidal thoughts and behaviors among individuals with mental disorders in Africa: a systematic review and meta-analysis of the past 25 years

**DOI:** 10.1186/s12888-025-07453-4

**Published:** 2026-02-12

**Authors:** Chaymaa Ghizlane, Imane Zougrar, Abir Benayad, Ossama Abdelaal, Oumnia Bouaddi, Imad Elbadisy, Byron Bitanihirwe, Saber Boutayeb, Lahcen Belyamani, Mathilde Husky, Mohamed Khalis

**Affiliations:** 1 Department of Public Health and Clinical Research, Mohammed VI Center for Research and Innovation, Rabat, Morocco; 2 Faculty of Medicine and Pharmacy of Rabat, Mohammed V University in Rabat, Rabat, Morocco; 3 Science for Africa Foundation, Nairobi, Kenya; 4 Mohammed VI International School of Public Health, Mohammed VI University of Sciences and Health, Casablanca, Morocco; 5 INSERM U1219, Bordeaux Population Health Research Center, Bordeaux University, Bordeaux, France; 6 Higher Institute of Nursing Professions and Health Techniques, Ministry of Health and Social Protection, Rabat, Morocco

**Keywords:** Suicidal thoughts and behaviors, Psychiatric disorder, Suicide ideation, Suicide attempt, Suicide, Africa, Mental health

## Abstract

**Background:**

Suicide causes over 800,000 deaths globally each year, with 73% occurring in low- and middle-income countries (LMICs). However, data on suicidal thoughts and behaviors (STBs) among individuals with psychiatric disorders in Africa remain limited. This study aims to assess the prevalence of STBs in this population using both general and clinical samples, addressing key data gaps in the African context.

**Methods:**

This systematic review and meta-analysis searched PubMed, Web of Science, Scopus, and other databases from January 2000 to January 2025. Study selection followed PRISMA guidelines, with Rayyan and Zotero used for screening and duplicate removal. Data were extracted using a structured framework, and study quality was assessed with Joanna Briggs Institute tools. Statistical analyses, including I² heterogeneity evaluation, were conducted in R using a random-effects model.

**Results:**

Eighty-two studies (*n*= 24,687) were included in the final meta-analysis. Recent suicide attempts (SA) prevalence was at 9% (95% CI: 6–12%, I² = 91%): substance-related disorders (17%), bipolar (15%), depressive disorders (14%), and schizophrenia spectrum disorders (6%). Lifetime SA prevalence was at 24% (95% CI: 21–27%, I² = 95.3%): cluster B personality disorders (33%), substance-related disorders (28%), schizophrenia spectrum disorders (27%), bipolar disorders (25%), and trauma-related disorders (25%). Recent suicidal ideation (SI) was at 31% (95% CI: 25–37%, I² = 97.5%): bipolar (61%), trauma-related disorders (58%), anxiety disorders (44%), depressive disorders (36%), and schizophrenia spectrum disorders (20%). Lifetime SI was 40% (95% CI: 33–46%, I² = 98.4%): patients with schizophrenia (41%), bipolar disorders (40%), and depressive disorders (39%).

**Conclusions:**

STBs among African individuals with psychiatric disorders represent a serious public health concern. These findings are essential for guiding targeted mental health policies and refining global STBs estimates.

**Supplementary Information:**

The online version contains supplementary material available at 10.1186/s12888-025-07453-4.

## Introduction

Mental health disorders are global public health issues affecting an estimated 970 million individuals globally as of 2019, with anxiety and depressive disorders representing the predominant conditions according to the World Health Organization (WHO) [1]. Among the most concerning consequences of mental health disorders is suicide, which is defined as the deliberate act of taking one’s own life, accounting for approximately 800,000 deaths each year and ranking as the second leading cause of death among individuals aged 15–29 years [2]. Recognizing the severe health and socioeconomic repercussions of suicide, the United Nations’ Agenda for Sustainable Development Goals (SDGs) has prioritized suicide prevention; specifically, through its target 3.4, it calls for coordinated international efforts to reduce global suicide mortality by 2030, aiming to promote mental well-being and reduce premature mortality from noncommunicable diseases, including mental health conditions, by one-third [3]. However, progress toward these goals may be uneven. For instance, evidence shows that although low- and middle-income countries (LMICs) report lower suicide rates compared to high-income countries, they account for 75.5% of global suicide cases [2]. Some groups, such as individuals affected by severe mental disorders (e.g., schizophrenia, bipolar disorder (BD), and depression), are disproportionately affected. Indeed, suicide attempts have been linked to several key predictors, including a history of non-suicidal self-injurious behavior, previous suicide attempts, the presence of any personality disorder, and prior psychiatric hospitalization, all of which indicate heightened vulnerability to repeated self-harm [4]. Similarly, suicidal ideation is predicted by factors such as a prior history of suicidal thoughts, persistent feelings of hopelessness, a clinical diagnosis of depression, a history of substance abuse, and co-occurring anxiety disorders, underscoring the complex interplay of psychiatric conditions in STBs [4]. A worldwide review estimated that up to 20% of patients with schizophrenia attempt suicide, with a 10% fatality rate [5, 6]. Some reviews have shown a prevalence of suicide attempts of 20–60% and 17% among persons with BD and major depressive disorders (MDD), respectively, and likewise, suicidal ideation estimates are 79% (during the depression phase) and 56%, respectively [7–9].

In Africa, there remains a significant unmet need for psychiatric services due to competing priorities, resource constraints and insufficient mental health infrastructure [10]. In addition, access to and demand for services are limited by barriers such as stigma, lack of trained professionals, and limited integration of mental health services into primary care settings [11]. In this context, the prevalence of STBs is expected to increase due to poor access to mental health resources compounded by the persisting socioeconomic challenges in these regions [12]. Indeed, according to the WHO, approximately 11.5 people per 100,000 per year die by suicide in the African region, which is higher than the global average of 8.9 per 100,000 people and is due in part to insufficient action to address and prevent risk factors, including mental health conditions [13]. Similarly, a recent meta-analysis on suicide attempts across the African continent reported a prevalence of 9.9% [14, 15].

The prevalence of STBs in individuals with mental disorders is not well established in the African context. This may be due mainly to challenges in the accuracy and comprehensiveness of suicide data across African countries. While some countries have relatively well-established mental health research frameworks, others lack systematic reporting mechanisms, often leading to underestimation of suicide-related statistics. Many African nations do not have centralized suicide registries, and the classification of deaths is often unreliable due to stigma, religious and cultural factors, and limitations in forensic investigations [16]. Data recorded in health information systems often do not capture mental health-related aspects. Furthermore, the reliance on hospital records alone excludes cases occurring outside formal healthcare settings, and the criminalization of suicide in some countries also further discourages reporting. Owing to these challenges, critical information gaps persist regarding STBs among individuals diagnosed with mental disorders, despite their well-documented associations/relationships [17, 18]. These gaps limit our understanding of the differential burden of STBs among this group and the development of appropriate and tailored awareness, prevention and response interventions. Despite the underestimation of data, this systematic review and meta-analysis seeks to apply rigorous methodology and appropriate statistical tools to generate a well-supported pooled prevalences of suicidal thoughts and behaviors (including suicide attempts and suicidal ideation ) among individuals with mental health disorders in Africa, drawing on evidence from both population-based studies and clinical samples. Such prevalence data would assist policymakers, given the urgent need to understand the extent of STBs in this population to inform culturally competent mental health care and support the implementation of decriminalization policies across the continent. These measures are essential to better capture the true burden of suicide-related outcomes, improve access to care, and ultimately reduce the long-term economic and public health consequences. Addressing this knowledge gap is critical for informing policy and clinical priorities within psychiatric care in Africa.

## Methods

This systematic review and meta-analysis is reported according to the PRISMA 2020 guidelines [19]. We registered the protocol with PROSPERO [CRD: 42,024,498,123] and published it.

**Box 1:** Definition of concepts

Main outcomes were defined on the basis of National Institute of Mental Health (NIMH) descriptions. • A suicide attempt is a nonfatal, self-directed, potentially injurious behavior with the intent to die as a result of the behavior. A suicide attempt might not result in injury. • Suicidal ideation refers to thinking about (suicidal thoughts: active and passive), considering (suicidal intent), or planning suicide (suicide plan). Suicide communications: a superset category generated by consensus papers on suicide nomenclature (see reference 22 and 23) and it accounts for suicidal threat and suicide plan. Suicide related communication can include a suicide note (suicidal threat) or a systematic formulation of a program of action that can lead to self-injury (suicide plan). Suicidality: the American Psychological Association defines suicidality as “the risk of suicide, usually indicated by suicidal ideation or intent, especially as evident in the presence of a well-elaborated suicidal plan”. It can also be defined to include suicidal thoughts, plans, gestures, or attempts. Suicide risk: refers to an individual’s likelihood of dying by suicide. In situations requiring emergency intervention, the term ‘imminent suicide risk’ may also be used. (see reference 20).	

Suicidal risk or suicidality are hereafter referred to as “suicidal thoughts and behaviors (STBs)” [20, 21]. We use the terminology and definitions outlined in recent consensus papers on this topic [22, 23]. If a term related to STBs is used in a manuscript whose outcomes and definition in the corresponding study meet our inclusion and exclusion criteria, we adopt our standardized terminology for consistency. STBs are classified more specifically into three categories: suicide ideations, suicide communications (suicide threats and plans) and suicide behaviors (suicide attempts) (suicide attempts and suicidal ideations are evaluated as the main outcomes). In this study, STBs are specifically defined as involving suicidal intent. Most researchers and clinicians distinguish suicidal behavior from non-suicidal self-injurious thoughts and behaviors (e.g., self-cutting), which refer to self-injury in which a person has no intent to die; such behavior is not the focus of this systematic review.

### Inclusion and exclusion criteria

This review targeted all human participants, permanently residing in Africa and who were diagnosed with psychiatric disorders according to the DSM-5/DSM-IV/DSM-IV-TR or ICD-10, including subjects who were diagnosed by psychiatrists in a hospital setting (with data collected from chart reviews, study-developed questionnaires or interviews and validated assessment tools) and who reported point (2 weeks up to one month), current/recent (2 weeks, 1 month, 6 months or 12 months) or past/lifetime suicidal ideation and suicide attempt prevalences, regardless of sex, age, setting (institution/health facility/school and community), population type (patient, general population), and psychiatric disorder timeframe (lifetime, recent). The 54 African countries and psychiatric disorders considered are detailed in Supplementary material Table 1. The exclusion criteria included studies on migrants, refugees, internally displaced persons, stateless individuals, and nonhumans; research on non-suicidal self-injury, parasuicide, or self-harm without specified suicidal intent; and studies without suicide ideation or attempts as a primary or secondary outcome. Only studies published from 2000 onward were included; studies conducted and/or published before 2000 were excluded. The following study designs were included: cohort/longitudinal studies (whether prospective or retrospective, data were only extracted from baseline characteristics), cross-sectional studies (descriptive, analytical and/or comparative), and case-control studies. Case reports, case series, abstracts, editorials, letters, duplicates, commentaries, literature, consensus statements, opinions, historical articles, meta-analyses and systematic reviews were excluded. However, references from systematic reviews/meta-analyses were manually screened for eligible primary studies. For studies needing clarification, the authors were contacted via email.

### Search strategy and selection process

We searched PubMed/MEDLINE, Web of Science, Scopus, Google Scholar, and ScienceDirect for articles published from 1 January 2000 onward, with no language restrictions. Scoping searches were first conducted to refine the search terms, after which structured searches were launched in September 2023 using Medical Subject Headings (MeSH), title/abstract keywords, publication dates, and publication types, combined with Boolean operators (AND, OR, NOT). A population-independent, outcome (prevalence of suicide-related behaviors) and geographical area (Africa)-defined strategy was initially applied, followed by two population-centered strategies targeting psychiatric disorders classified under the DSM-IV, DSM-IV-TR Axis I and Axis II disorders and DSM-5. The full search strategies are detailed in the Supplementary Data. Searches were updated in February 2024, October 2024, and January 2025 to capture additional eligible studies. Records were imported into Rayyan [24], duplicates were removed, and titles and abstracts were screened independently by four reviewers (CG, IZ, OA, AB) using blind mode to minimize bias. Studies meeting the inclusion criteria were retained. Conflicts were resolved through discussion after the blind mode was deactivated. The full texts were subsequently screened via Zotero [25]. Updates to the search strategy were followed by second, third, and fourth rounds of screening in Rayyan, again using blind mode and involving two to three reviewers per round (CG, OA, IZ). Records where outcomes or populations of interest were reported as secondary findings were also included. For inaccessible full texts, the corresponding authors were contacted via email or ResearchGate. To ensure the comprehensiveness and relevance of the included articles, a co-author (MH), an expert in psychology with research experience in suicidology, reviewed both the included studies and the search strategy. Additionally, the supervisory team contributed to maintaining the overall quality of the search and screening processes.

### Data items and collection process

Data extraction was conducted via a structured Microsoft Excel sheet, which included the following: lead author and coauthors listed as “et al.”, publication date, country of study, study setting (community, institution) and patient setting (outpatients and/or inpatients), study years, study design, sampling technique, sample size along with comprehensive participant demographics (including female proportion, age range, age mean ,and standard deviation), sample definition and psychiatric disorder assessment tools and/or disorder classification system for the psychiatric condition, suicide ideation and suicide attempt-related information (assessment tool, cases and timeframe of the measured prevalence). Articles written in a language other than English were also translated.

The lead author independently collected data from the eligible reports, and a second reviewer verified the extracted information. To ensure reliability and validity, a third reviewer oversaw the data extraction information during the quality assessment phase, along with the lead author. Clarifications were sought from the study authors when necessary. Data adjustments included the following:

- For studies treating ‘suicidal thoughts,’ ‘suicidal intent,’ and ‘suicide plan’ as distinct, nonoverlapping outcomes (e.g., subjects with a suicide plan are not counted under suicidal thoughts), we combined these variables under the broader category of ‘suicidal ideation’. However, in studies where these outcomes were not distinctly separate (i.e., subjects with suicidal ideation also included those with suicidal plans or intent, while the latter were also reported separately), we considered only the ‘suicidal ideation’ variable, which encompassed both suicidal plans and intent.
- If the mean age (M±SD) of participants was provided separately for subsets of comparison groups rather than for the full sample, each characteristic of the full sample was derived by calculating the relevant statistics from the two subsets. The percentages were also converted into absolute numbers for statistical analysis.
- If a study included multiple psychiatric disorders (whether specified or not) without distinguishing outcomes for each specific disorder (regardless of comorbidities among them), the overall outcome was attributed to the combined group. For example, in a study on severe mental disorders (SMDs), such as major depressive disorder (MDD), bipolar disorder, and schizophrenia, if suicide attempts were reported only for the total sample rather than for each disorder separately, the study was categorized under the subgroup ‘Different Psychiatric Disorders’.

### Quality appraisal

Two reviewers independently appraised the quality of the studies via the Joanna-Briggs Institute (JBI) tools for cross-sectional and case‒control studies [26]. The checklist contains a total of 8 assessment criteria for cross-sectional studies and 10 items for case‒control studies with response options that include yes, no, unclear, and “not applicable”. The results of each individual paper were graded with a score ranging from 0 to 8 for cross-sectional studies and from 0 to 10 for case-control studies. Inter-rater scoring discrepancies during critical appraisal were resolved after a thorough discussion, and if disagreement persisted, the quality assessment score was determined on the basis of the calculated mean scores of the two reviewers. All the articles that scored 50% or greater were included in the analysis.

### Data analysis

The pooled prevalence of STBs and the corresponding 95% confidence intervals (CIs) were calculated via a random-effects model. Study heterogeneity was evaluated by the I^2^ statistic, with an I^2^ greater than 50% indicating high heterogeneity [27]. A subgroup analysis was performed to explore the source of heterogeneity. Subgroup analyses were performed based on the following categorical variables: individual setting, sampling technique, study country, study quality, and measurement instrument for SI/SA, and they only included studies employing a cross-sectional design. A sensitivity analysis was conducted to test the consistency of the primary results by removing each study one by one. The significance level was set as *p* < 0.01. Data analyses were conducted with R version 4.4.2 via the Metaprop function. Publication bias was examined via funnel plots and Egger’s test [28].

## Results

**Fig. 1:**
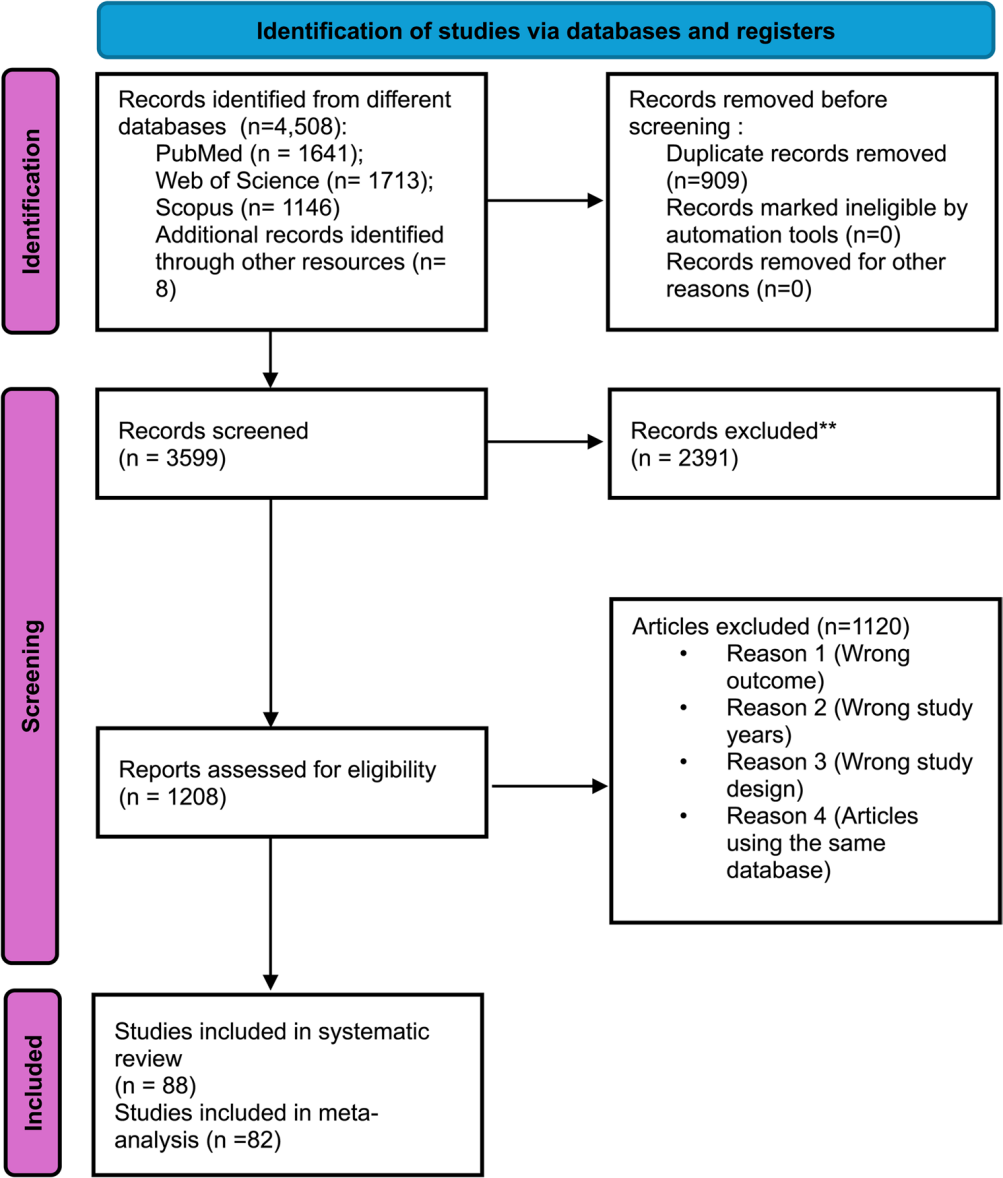
PRISMA diagram flow for study selection

The PRISMA 2020 flow diagram of the studies selected and included in the systematic review and meta-analysis is provided in Fig. 1. From our database search, 4508 studies were primarily identified. As a result of duplication, 909 studies were removed. A total of 1208 full-text studies were identified, and 1120 studies were removed for not meeting our inclusion criteria. After all the exclusions, a total of 88 studies covering 25,568 persons living with psychiatric disorders and fulfilling the eligibility criteria for the study were included in the systematic review. The final meta-analysis included 82 studies–covering 24,687 individuals–excluding those with selection bias due to non-cross-sectional study designs.

The study characteristics are presented in Table 1. The review included participants from 10/54 African countries covering 26% of the continent’s geographic area, with 22 (25%) studies from Ethiopia, 13 (14.78%) from Morocco and South Africa each, 12 (13.63%) from Tunisia, 11 (12.5%) from Nigeria, 10 (11.36%) from Egypt, 3 (3.40%) from Uganda, 2 (2.27%) from Kenya and 1 (1.14%) from Sudan and Malawi each. The sample sizes of the 88 studies ranged from 12 to 3104, and the mean age was available from 63 studies and ranged from 22 ± 9.73 to 46.8 ± 13.2 years, with the age gap ranging from 10 to 99 years. Age ranges were available for 74/88 studies, of which 61 studies (82.43%) included only individuals above the age of 18. Among the studies, 82 (93.18%) were cross-sectional, 4 (4.54%) were cohort studies, and 2 (2.27%) were case-control studies. The sex proportion was available for 78 (88.63%) studies in which the number of studies including male proportions equal to or above 50% was 51 (65.38%). The individuals settings were reported in all studies, with 59 studies (67.04%) involving outpatients, 19 studies (21.59%) involving inpatients, 8 studies (9.09%) including both outpatients and inpatients and 2 studies (2.27%) that were community-based. Most studies used non-probability sampling techniques (*n* = 59/84, 70.23%), and most studies reporting psychiatric disorder classification systems (ICD-10 or DSM) used the DSM criteria (*n* = 61/65, 93.84%). The rates of STBs associated with various psychiatric disorders were presented in both single and separate studies. Overall, 65 and 28 studies reported lifetime prevalence rates of suicide attempts and ideation, respectively, whereas 15 and 26 studies reported recent prevalence rates of suicide attempts and ideation, respectively. All the studies were conducted and published between 2000 and 2025, except for 16 studies published after 2000 with unknown study years. Given the high heterogeneity observed from the variance between the included studies, a subgroup analysis based on the DSM-defined psychiatric disorder classification was employed. A subgroup analysis by individual setting, sampling technique, study country, study quality, and measurement instrument for SI was also performed but could not identify the source of heterogeneity (Supplementary material SM4).

**Table 1:** Study characteristics

Authors Year Country	Study years	Study design	Study setting	Study participants	Female proportion (%)	Age M and SD/Age range	Individuals setting	Psychiatric disorder assessment tools and/or disorder classification system	Screening tool for SI/SA
Allan et al. [29] 2001 South Africa	NR	Cross-sectional	Alcohol rehabilitation center	260 Subjects with alcohol abuse or dependence	15.98%	38.4 ± 8.43	Inpatients	Study semi-structured questionnaire	Study semi-structured questionnaire
Ibrahim et al. [30] 2022 Egypt	March 2020-February 2021	Cross-sectional	Psychiatric outpatient clinic	Outpatients with substance abuse disorder related to pregabalin : 83. Co-MDD: 62, Co-GAD: 39, Co-BPD : 65, Co-APD : 31	3.6%	32.61 ± 7.06 20–56	Outpatients	SCID-I. Right Sign screen test (urine screening test)	BSS
Aboubacar et al. [31] 2022 Morocco	September 2019-March 2020	Cross-sectional	University psychiatric hospital	115 Patients with schizophrenia	22.6%	30.9 ± 0.83 16–62	Inpatients	FTND/FTQ/CAST/AUDIT/ECAB/Study questionnaire	Study questionnaire
Bout et al. [32] 2022 Morocco	NR	Cross-sectional	University psychiatry department	32 Patients with psychogenic nonepileptic seizures	93.75%	29.13 ± 9.6 18–61	Outpatients	MINI; Electrodiagnosis (Video EEG)	MINI
Obadeji et al. [33] 2014 Nigeria	January-June 2009	Cross-sectional	Teaching hospital psychiatry unit	30 PLWHA and MDD	83.3%	39.20 19–64	Outpatients	SCID I	SCID I/HDRS
Abdel Hamid et al. [34] 2019 Egypt	March 2014-February 2015	Cross-sectional	University addiction psychiatry clinic	90 Patients with bipolar I disorder	47.7%	31.97 ± 9.54 18–65	Outpatients	SCID I	BSS
Kachouchi et al. [35] 2018 Morocco	January 2005 −31st August 2015	Case‒control	University psychiatry department	120 Patients with schizophrenia	6.7%	32.8075 ± 9.139	Inpatients	DSM-IV	Interviews with patients & medical records
Ferreira-Correia et al. [36] 2020 South Africa	NR	Cross-sectional	University genetics division	13 Patients with HD	46%	46.8 ± 13.2 32–68	Outpatients	Genetically confirmed UHDRS for disease progression and clinical performance	UHDRS
Sharaf et al. [37] 2012 Egypt	NR	Cross-sectional	Regional outpatient psychiatric clinic	200 Patients with schizophrenia	16.5%	30.40 ± 6.82 17–48	Outpatients	DSM-IV-TR	SPS; Study structured interview
Negash et al. [38] 2005 Ethiopia	NR	Cross-sectional	District health centers	289/290 Cases with bipolar I disorder	44.7%	15–49	Community-based	CIDI SCAN/DSM-IV	CIDI SCAN
Mechri et al[39] 2011 Tunisia	NR	Cross-sectional	University psychiatry department	98 Patients with recurrent depressive disorder	56.1%	46.8 ± 9.9 22–65	Outpatients	DSM-IV-TR/psychiatric Interview	Study questionnaire, psychiatric and medical examination, medical records
Getnet et al. [40] 2022 Ethiopia	February 01st-Match 01st 2019	Cross-sectional	Regional general hospitals	610 Patients with mental disorders	52.3%	31 ≥ 18	Outpatients	ISMI/OSS/Study questionnaire	A structured questionnaire
Amamou et aI [41] 2018 Tunisia	January 2008-December 2015.	Cross-sectional	University psychiatry department	173 Patients with bipolar I disorder	31.8%	43.3 ± 12.2	Inpatients	DSM-5/psychiatric interview	Interviews with patients (preestablished questionnaire) & medical records review
Duko et al. [42] 2018 Ethiopia	August–September 2016.	Cross-sectional	Specialized psychiatric hospital	270 Patients with schizophrenia and 272 bipolar disorder	29.52%	schizophrenia: 33.71 ± 9.35 BD : 32.62 ± 9.43 20–53	Outpatients	SCID-IV	CIDI
Oladeji et al. [43] 2017 Nigeria	March-August 2011	Cross-sectional	University HIV clinic	66 PLWHA, Depression and or GAD	NR	41.3 ± 10.0 18–70	Outpatients	CIDI/DSM-IV	CIDI
Kibru et al. [44] 2020 Ethiopia	May–June 2018	Cross-sectional	Specialized psychiatric hospital	409 Patients with schizophrenia	37.7%	22 ± 9.73 ≥ 18	Outpatients	OSS/PANSS/LSAS-SR	CIDI
Bram et al. [45] 2012 Tunisia	January 1 st,2000-December 31,2006	Cohort	University psychiatry department	38 Patients with bipolar disorders I + II	39.4%	37.81	Inpatients	DSM IV TR	NR
Barnett et al. [46] 2018 Malawi	January,01st- December 31st, 2011	Cross-sectional	Regional psychiatric unit	419 Inpatients with mental disorders	27.1%	29.6 ± 9.5 10–74	Inpatients	ICD-10	NR
Ndetei et al. [47] 2008 Kenya	June 2004	Cross-sectional	Mathari Psychiatric Hospital Nairobi	170 Patients with MDD	38.8%	≥ 11	Inpatients	Patients files, SCID-I	SCID-I
Ndetei et al. [48] 2009 Kenya	June 2004	Cross-sectional	National psychiatric hospital	Psychiatric patients. 691.Substance abuse : 42 Psychosis : 138 BD-I: 159 Schizo-affective : 29 schizophrenia : 234	36.9%	NR	Inpatients	SCID I. review of medical records	SCID-I
Assefa et al. [49] 2012 Ethiopia	NR	Cross-sectional	Specialized psychiatric hospital	212 Patients with schizophrenia	34.9%	33.3 ± 8.9 ≥ 18	Outpatients	Medical records review and clinical diagnosis, DSM-IV	CIDI single question on suicide attempts
Niehaus et al. [50] 2004 South Africa	NR	Cross-sectional	Hospital inpatient and community clinics	454 Xhosa individuals with schizophrenia	23.1%	11–35	Inpatients	DIGS	DIGS
Du Toit et al. [51] 2020 South Africa	April 2011-July 2017	Cross-sectional	Maternal mental health outpatient clinics	280 Pregnant women with a psychiatric illness	100%	31 18–46	Outpatients	DSM-IV-TR/Psychiatric interview	MINI; MADRS
Basha et al. [52] 2021 Ethiopia	March 01st–30th 2019	Cross-sectional	Specialized psychiatric hospital	337 Patients with MDD	55.5%	31.82 ± 10.14 ≥ 18	Outpatients	OSS/Study questionnaire	CIDI
Adayonfo et al. [53] 2017 Nigeria	January 2010-December 2015	Cross-sectional	University psychiatric outpatient clinics	152 Patients with Depression	55.3%	12–99	Outpatients	ICD-10/Psychiatric interview	ICD-10
Kinyanda et al. [54] 2011 Uganda	6th May 2010-10th August 2010	Cross-sectional	Government HIV clinics	50 PLWHA and MDD	9.6%	≥ 19	Outpatients	MINI	MINI
Fekih-Romdhane et al. [55] 2019 Tunisia	January-June 2017	Cross-sectional	University psychiatric services	108 Patients with bipolar I disorder	36.1%	41.8 ± 12.2 18–65	Outpatients	DSM-5; YMRS; HDRS-17	The third item of HDRS-17
Vilyte et al. [56] 2024 South Africa	2008–2022	Case‒control	Private hospital neuroscience unit	305 Patients with psychogenic nonepileptic seizures (PNES)	77.4%	NR	Outpatients	Electrodiagnosis (Video EEG), International League Against Epilepsy (ILAE) FS criteria	Patients’medical records
Kassie et al. [57] 2022 Ethiopia	5th May- 13th June 2019	Cross-sectional	Specialized and teaching hospitals	408 Outpatients with substance use disorder	13.7%	18–70	Outpatients	Study questionnaire/OSS/SAPSS	CIDI
Ahmed et al. [58] 2021 Egypt	November 2017–December 2019	Cohort	University psychiatric unit	216 Patients with Bipolar I disorder	34.3%	31.67 ± 7.35 18–50	Inpatients	SCID	NR
Getahun et al. [59] 2023 Ethiopia	April 01st-July 30th 2022	Cross-sectional	Specialized teaching hospitals	423 People with severe mental disorders	34.1%	33.8 ± 10.753 18–65	Inpatients	DSM-5	CSSRS; chart review
Hasan et al. [60] 2024 Egypt	2021–2022	Cross-sectional	University psychiatry institute	120 Patients with MDD	79.2%	28 ± 8 18–56	Outpatients	DSM-IV Axis I Disorders (SCID-I)	C-SSRS
Najim et al. [61] 2015 Sudan	June 2005- June 2010	Cross-sectional	Private psychiatric clinic	137 patients with depression	41.6%	NR	Outpatients	ICD-10	NR
Bosman et al. [62] 2020 South Africa	NR	Cross-sectional	Forensic psychiatric hospital	37 Male State patients with ASPD or antisocial personality traits	0%	NR	Inpatients and outpatients	Study questionnaire	BSS
Koubaa et al. [63] 2023 Morocco	February 2021-February 2022	Cross-sectional	Psychiatric hospital department	304 Patients with schizophrenia	22.4%	37.3 18–68	Inpatients	DSM-5/PANSS/insight scale Q8/MARS	A detailed questionnaire addressed to patients their families; medical records.
Féki et al. [64] 2019 Tunisia	September-November 2016	Cross-sectional	Chronic disease outpatient clinic	17 Elderly patients with type 2 diabetes and symptoms of depression	NR	65–88	Outpatients	GDS	GDS
Adeosun et aI [65] 2013 Nigeria	January-April 2012	Cross-sectional	National psychiatric hospital	246 Patients with PMD and NMD	65.8%	39.89 ± 13.36 ≥ 18	Outpatients	SCID I for DSM-IV; HAM-D	SCID-I
Doufik et al. [66] 2024 Morocco	April 1 st,2020-30th June 2021	Cohort	University medical faculty	102 Patients with schizophrenia or schizoaffective disorder	18.6%	39 ± 10.5 18–65	Outpatients	DSM-5/PANSS/CGI/CDS/MARS	NR
Bantjes et al. [67] 2017 South Africa	NR	Cross-sectional	Community HIV testing centers	220 Individuals with psychiatric disorders seeking HIV testing. Depressive disorder : 87 GAD : 15 Trauma and stress related disorders : 26 AUD : 92	NR	18–71	Outpatients	SCID	**BDI**
Dereje et al. [68] 2024 Ethiopia	October 15th-November 15th, 2022.	Cross-sectional	Specialized and general hospitals	402 Adult individuals with mental illness	43.8%	34.65 ± 10.56 18–64	Outpatients	DSM-5 (SCID 5)	SBQ-R
Moussaoui et al. [69] 2024 Morocco	2020–2021	Cross-sectional	University psychiatry department	206 Patients with bipolar disorder I and II	41.7%	37.34 ± 12.53 17–70	Inpatients and outpatients	DSM-5//FTND/CAST/AUDIT/ECAB	Psychiatric interview/Study questionnaire
Ben Thabet et al. [70] 2024 Tunisia	June-August 2016	Cross-sectional	Primary healthcare centers	78 Patients with GAD	13.3%	42.2 ± 16 ≥ 18	Outpatients	MINI	NR
Abaatyo et al. [71] 2023 Uganda	2018–2021	Cross-sectional	Mental health inpatient unit	3104 individuals with severe mental conditions	44%	33 ± 14.0	Inpatients	DSM-5	Medical records
Peltzer et al. [72] 2013 South Africa	May-October 2011	Cross-sectional	Public primary care clinics	2541 TB patients with Psychiatric disorders. PTSD : 1441. AUDIT : Hazardous 785 AUDIT : Harmful alcohol use : 315	NR	18–93	Outpatients	PC-PTSD, AUDIT	Study questionnaire
Habtamu et al. [73] 2019 Ethiopia	February 03rd-December 11th 2015	Cross-sectional	District health centers	92 Individuals with depression	71.7%	38.1 ± 14.1 ≥ 18	Outpatients	PHQ-9 & PHC-staff diagnosis	CIDI
Tamirat et al. [74] 2021 Ethiopia	November-January 2019	Cross-sectional	Regional referral hospital	80 PLWHA and depression	NR	≥ 18	Outpatients	PHQ-10	CIDI
Viswasam et al. [75] 2017 South Africa	2000–2016	Cross-sectional	Specialized psychiatric centers	565 Patients with obsessive-compulsive disorder (OCD)	50.1%	32.2 ± 13.0	Outpatients	SCID I; MINI	NR
Sori et al. [76] 2022 Ethiopia	February 01st-July 01st 2021	Cross-sectional	University psychiatric clinic	365 Patients with mental illnesses	47.1%	34.96 ± 11.26 ≥ 18	Outpatients	ISMI/OSS/RSES/MARS/ASSIST/Study questionnaire and psychiatric interviews	CIDI
Zewdu et al. [77] 2021 Ethiopia	February- June 2019	Cross-sectional	Maternal HIV prevention clinics	159 Perinatal WLWHIV with depression	100%	≥ 18	Outpatients	EPDS	CIDI
Anyayo et al. [78] 2021 Uganda	April 15th-June 20th 2017	Cross-sectional	Regional mental health clinic	169 Patients with Bipolar disorder	54.4%	37.2 ± 12.8 ≥ 18	Outpatients	Clinical diagnosis	Study questionnaire
Chakroun et al. [79] 2022 Tunisia	October 01st2018-May 01st 2019	Cross-sectional	University psychiatric services	150 Patients with bipolar I disorder. Anxiety disorder : 26 Personality disorder : 18 (Histrionic: 10, Antisocial : 6) SUD : 49”	NR	45.21 ± 11.19 ≥ 18	Outpatients	DSM-5 HADS; YMRS	NR
Luckhoff et al. [80] 2014 South Africa	NR	Cross-sectional	Hospitals and community clinics	974 Xhosa individuals with schizophrenia and Schizoaffective Disorder (25)	19.5%	35 ± 10.5	Inpatients and outpatients	DIGS; DSM–IV TR	DIGS
Scholtz et al. [81] 2005 South Africa	NR	Cross-sectional	Public and military hospitals	197 Afrikaner patients with : bipolar disorder (I-II): 74, schizo-affective disorder : 43 and schizophrenia: 80	39.6%	BD : 44.5 ± 13.6 Schizo-affective : 33.7 ± 12.1 Schizophrenia : 31.3 ± 11.4	Inpatients and outpatients	DIGS; DSM–IV	DIGS; DSM–IV
Bassiony et al. [82] 2022 Egypt	January 2016-December 2017	Cross-sectional	University psychiatry services	100 Patients with SUD	7%	30.71 ± 8.68 18–57	Inpatients and outpatients	DSM-IV-TR; SCID I; ASI (for drug related problems)	BSS
Mekonnen et al. [83] 2011 Ethiopia	March-December2006	Cross-sectional	University psychiatric clinic	474 Psychiatric patients	46%	32 ± 12.3 18–82	Outpatients	DSM-IV	Prepared Structured questionnaire
Ammar et al. [84] 2008 Tunisia	March 01st-30th June 2003	Cross-sectional	University psychiatry consultation	360 patients with depression	84.7%	45.8 18–65	Outpatients	DSM-IV//SF-36	NR
Sehlo et al. [85] 2022 Egypt	March-December 2019	Cross-sectional	University psychiatric clinic	160 Women with Depression	100%	37.22 ± 6.50	Outpatients	SCID-5-CV	SSI
Sehlo et al. [86] 2021 Egypt	January-October 2019	Cross-sectional	University psychiatry department	120 Patients with obsessive-compulsive disorder (OCD). Co-MDD : 37 Co-GAD : 22 Co-Panic disorder : 3”	50%	36.24 ± 7.21 18–60	Outpatients	SCID-I-CV	SSI
Ayalew et al. [87] 2021 Ethiopia	March 01st–30th 2019	Cross-sectional	Specialized psychiatric hospital	300 Patients with Schizophrenia	32.3%	≥ 18	Outpatients	DSM-5/CDSS/OSSS	SBQ-R
Barrimi et al. [88] 2021 Morocco	February 2019	Cross-sectional	Medical faculties	178 Medical Students with mental disorders	NR	16–35	Community-based	DSM-5	NR
Barrimi et al. [89] 2023 Morocco	August 2019-July 2021	Cross-sectional	University psychiatry department	400 Women with severe psychiatric disorders	100%	44.64 ± 13.3 18–80	Inpatients	DSM V/FTND/CAST/AUDIT/ Psychiatric interviews and reviewing of medical records	Review of the medical record and clinical interviews with patients and available relatives
Barrimi et al. [90] 2023 Morocco	January-July 2016	Cross-sectional	University psychiatry department	250 Patients with a psychiatric disorder	22%	34 15–68	Inpatients	Psychiatric interview	Interviews with patients and their families patients medical files
Umar et al. [91] 2017 Nigeria	March 2013-December 2015	Cross-sectional	Teaching hospital rehabilitation unit	233 Adult patients with substance use disorder with and without ADHD diagnosis. ADHD : 50 no-ADHD : 183”	17.2%	26.31 ± 6.53 ≥ 18	Inpatients and outpatients	SSADDA 6.1; FTND; ASRS; DIVA 2.0 based on DSM-IV	NR
Jemal et al. [92] 2022 Ethiopia	July 15th-September 14th 2021	Cross-sectional	University medical center	401 Patients with mental illnesses	36.2%	34.69 ± 10.94 ≥ 18	Outpatients	Medical records/(PDQ-4 +) : DSM-5	Study questionnaire
Ogundipe et al. [93] 2015 Nigeria	NR	Cross-sectional	University HIV clinic	12 PLWHA and psychiatric illness	NR	20–63	Outpatients	Study questionnaire	BDI
Ogunnubi et al. [94] 2022 Nigeria	NR	Cross-sectional	University psychiatric outpatient clinic	160 Patients with schizophrenia	53.8%	38.54 ± 11.30 20–71	Outpatients	MINI 6	MINI 6
Aloba et al. [95] 2015 Nigeria	July 2013- June 2014	Cross-sectional	University psychiatric clinics	327 Patients with psychiatric disorders	58.1%	41.39 ± 12.97 ≥ 18	Outpatients	MINI/DSM-5	MINI
Esan et al. [96] 2021 Nigeria	February 23-October 10 2018	Cross-sectional	University and state psychiatric outpatient clinics	215 Patients with schizophrenia	49.3%	38.8 ± 9.4 20–60	Outpatients	DSM IV; SCID-I	CIDI
Esan et al. [97] 2022 Nigeria	February-October 2018	Cross-sectional	Teaching and state hospital psychiatry clinics	76 Patients with bipolar I disorder	61.8%	39.09 ± 11.10 18–60	Outpatients	DSM IV; SCID-I	NR
El Oumary et al. [98] 2024 Morocco	June 01st-August 30th, 2022	Cross-sectional	Provincial hospital psychiatry services	324 Patients with Mood disorders	66.7%	40.3 ± 10.8 18–66	Outpatients	MINI	SIS, MINI
Bouchra et al. [99] 2017 Morocco	February-July 2015	Cross-sectional	University psychiatric hospital	22 Patients with bipolar II disorder	82%	37.3 ± 7 ≥ 18	Outpatients	MINI	MINI
Chlihfane et al. [100] 2022 Morocco	August 2019-July 2021	Cross-sectional	University psychiatry department	204 Patients with psychiatric disorders	100%	42,90 ± 13,23 17–85	Inpatients and outpatients	DSM-5	Study questionnaire, interview with patients and their families
Mensi et al. [101] 2016 Tunisia	April 2013–March 2014	Cross-sectional	University psychiatry department	126 Patients with schizophrenia	27.8%	43.44 ± 10.60 20–65	Inpatients	DSM-IV/PANSS/CGI/EGF/BPRS/CALGAGY	Calgary Depression Scale for Schizophrenia
Khouadja et al. [102] 2024 Tunisia	January 01 st,2014-December 31 st, 2022	Cross-sectional	Regionaal psychiatry department	129 Patients with adjustment disorder	75.2%	14–72	Inpatients	DSM-5	NR
El Jabiry et al. [103] 2022 Morocco	October-December 2019.	Cross-sectional	University psychiatry department	187 Patients with schizophrenia. PTSD : 26 no-PTSD : 161	24%	41.45 ± 13.27 18–88	Inpatients and outpatients	MINI	MINI
Erfan et al. [104] 2010 Egypt	NR	Cross-sectional	Substance use disorder treatment unit	60 Male patients with SUD. SUD only : 30, SUD + depressive disorder : 30	0%	18–45	Inpatients	DSM-IV//ASI/HRSD/psychiatric interviews	HDRS
Naidoo et al. [105] 2017 South Africa	2015	Cross-sectional	Public, private, and university psychiatric services	239 Patients with psychiatric disorders	NR	18–68	Outpatients	DSM-5	BSS
Bouhlel et al. [106] 2013 Tunisia	March-April 2010	Cross-sectional	University psychiatry follow-up clinic	134 Patients with schizophrenia	38%	40.65 ± 11.64 21–79	Outpatients	DSM-IV/PANSS/SAS	**Interviews** with patients and their families patients medical files
Seid et al. [107] 2020 Ethiopia	November-January 2019	Cross-sectional	HIV therapy clinic	79 PLWHIV and depression	64.5%	≥ 18	Outpatients	PHQ-9	PHQ-9
Oriji et al. [108] 2020 Nigeria	NR	Cross-sectional	National psychiatric clinics	153 Patients with schizophrenia	48.4%	18–65	Outpatients	ICD-10; MINI 5	MINI
Abraham et al. [109] 2025 Ethiopia	June 15th - August 15th, 2021	Cross-sectional	Teaching and referral psychiatric clinic	306 Patients with psychiatric disorders	44.8%	33 ± 11.5 18–71	Outpatients	DSM-5/OSS-3/EUROHIS-QOL index/MGL MAQ/RSES	Study questionnaire/Interview
Fanta et al. [110] 2020 Ethiopia	October 2017-March 2018	Cross-sectional	Specialized psychiatric hospital	418 Patients with schizophrenia	31.3%	35.50 ± 9.25 ≥ 18	Outpatients	SCID I; DSM-IV-TR	PHQ-9
Hassan et al. [111] 2020 Egypt	March 01st-December 31st, 2015	Cohort	University psychiatry unit	100 Patients with psychiatric disorders	46%	30.34 ± 10.78	Inpatients	DSM-5/DRS	(S-STS)/SIS-MAP
Alemu et al. [112] 2024 Ethiopia	2023	Cross-sectional	University specialized hospital	636 People with mental illness	50.2%	35.5 ± 11.7 ≥ 18	Outpatients	RSES/WHOQOL-BRFE/MARS/ASSIST/CGI/Psychiatric interview	Interviewer administered questionnaire
Thungana and al [113] 2022 South Africa	September 2020 -June 2021	Cross-sectional	District primary care clinics	74 TB patients with Unipolar depression	NR	≥ 18	Outpatients	DSM-5/MINI	MINI
Hussien et al. [114] 2014 Ethiopia	November 2011-May 2012	Cross-sectional	Specialized psychiatric hospital	410 Patients with schizophrenia	40%	33.8 ± 10.753 ≥ 18	Outpatients	Clinical diagnosis	CIDI
Weret et al. [115] 2014 Ethiopia	April 01st-April 25th 2011	Cross-sectional	Specialized psychiatric hospital	400 Patients with schizophrenia	34.5%	15–79	Outpatients	Study questionnaire and psychiatric interviews	structured questionnaires by interview and record review technique
Abbes et al. [116] 2024 Tunisia	January 01st-July 30th, 2021	Cross-sectional	University psychiatric consultation clinic	133 Patients with psychiatric disorders	39.8%	45.02 ± 11.8 ≥ 18	Outpatients	DSM-5	SBQ-R

*NR* Not reported, *MDD* major depressive disorder, *PMD* psychotic major depression, *NMD* non-psychotic major depression, *SCID* Structured Clinical Interview for the DSM-IV, *MINI* Mini-International Neuropsychiatric Interview, *DSM* Diagnostic and Statistical Manual of Mental Disorders, *CIDI* Composite International Diagnostic Interview, *SCAN* Schedules for Clinical Assessment in Neuropsychiatry, *ICD-10* International Classification of Disease-10 (ICD-10), *WERCAP* The Washington Early Recognition Center Affectivity and Psychosis, *DIS* Diagnostic Interview Schedule for DSM-5 diagnosis, *DIGS* Diagnostic Interview for Genetic Studies, *YMRS* Young Mania Rating Scale, *HDRS‐17* Hamilton Depression Rating Scale, *PHQ-9* Patient Health Questionnaire, *PHC* primary health care, *EPDS* Edinburgh Postnatal Depression Scale, *CAGE* Cut down Annoyed Guilty and Eye-opener, *SSADDA 6.1* Semi-structured Assessment for Drug Dependence and Alcoholism Version 6.1, *FTND* Fagerström test for nicotine dependence, *ASRS* Adult ADHD Self-Report Scale, *DIVA* Diagnostic Interview for ADHD in Adults, *OCI* Obsessive–Compulsive Inventory, *OCS* Obsessive‒compulsive symptoms, *SSI* Scale for suicide ideation, *SPS* Suicide Probability Scale, *SBQ-R* Shona Suicidal Behavior Questionnaire-Revised, *BSS* Beck scale for suicide ideation, *CSSRS* Colombia Suicide Severity Rating Scale, *MADRS* Montgomery-Asberg Depression Rating Scale, *CTRS* crisis triage rating scale, *UHDRS* Unified Huntington's Disease Rating Scale, *HAM-D/HDRS* Hamilton Depression Rating Scale, *(S-STS)* Sheehan suicidality tracking scale, *ASI* Addiction Severity Index, *OSSS/OSS* Oslo Social Support Scale, *LSAS-SR* Liebowitz Social Anxiety Scale Self-Reporting, *ISMI* Internalized stigma of mental illness, *FTQ* Fagerström tolerance questionnaire, *ASSIST* Alcohol, Smoking and Substance Involvement Screening Test, *SAPSS* Abuse Perceived Stigma Scale, *RSES* Rosenberg self-esteem scale, *MARS* Medication Adherence Reporting Scale, *SAS* Simpson Angus rating scale, *MGL MAQ* The Morisky, Green, Levine Medication Assessment Questionnaire, *ECAB* Cognitive Scale of Attachment to Benzodiazepines, *CAST* cannabis abuse screening test, *AUDIT* Alcohol Use Disorders Identification Test, *EPSE* Extrapyramidal Symptoms Rating Scale, *SF-36* 36-Item Short Form Survey (SF-36) for measurement of quality of life , *PANSS* Positive And Negative Syndrome Scale, *GDS* Geriatric Depression Scale, *BDI* Beck's Depression Inventory, *CDS* Calgary Depression Scale, *CDSS* Calgary Depression Scale for Schizophrenia, *SIS-MAP* Scale for Impact of Suicidality- Management, Assessment and Planning of Care, *EUROHIS* *Qol Index* EUROpean HISpanics quality of life index, *MGL MAQ* The Morisky, Green, Levine Medication Assessment Questionnaire, *RSES* Rosenberg Self-Esteem Scale, *CGI* Clinical Global Impression, *DRS* Disability Rating Scale, *WHOQOL-BRFE* World Health Organization Quality Of Life Brief, *GAD* Generalized Anxiety Disorder, *BPD*: Borderline Personality Disorder , *PTSD* Post-Traumatic Stress Disorder, *APD/ASPD* Antisocial Personality Disorder , *AUD *Alcohol Use Disorder, *SUD* Substance Use Disorder, *EEG* Electroencephalogram , *PLWHA* People Living With Human immunodeficiency virus (HIV)/AIDS, *WLWHIV* Women Living With HIV, *HD *Huntington's Disease, *TB *Tuberculosis.

We assessed the quality of each study using the *Joanna Briggs Institute (JBI)* Critical Appraisal Checklist for observational cohort, cross-sectional, and case-control studies [26]. This checklist evaluates key aspects, including sample selection, consistency in data collection, and control of confounders, allowing us to gauge the reliability of each study. A score on this checklist of 7 and above for cross-sectional and cohort studies was considered high quality, a score of 4 to 6 was considered moderate quality, and a score less than 4 was considered low quality. For case-control studies, a score of 8 and above was considered high quality, a score of 5 to 7 was considered moderate quality, and a score of less than 5 was considered low quality. Most studies (53.40%) were rated as high quality in accordance with the JBI criteria. These studies showed strong methodological rigor, including clear sampling, valid measurement of variables, and thorough control of biases, resulting in a high level of confidence in their findings. The remaining studies were rated as moderate quality, meaning that they generally met important criteria but had minor limitations that could introduce some bias. Importantly, no studies were rated as low quality, suggesting that all studies included here met a reliable threshold for methodological soundness. For further detail, *Supplementary material Table *2 provides a breakdown of each study’s quality rating and specific checklist criteria.

**Fig. 2:**
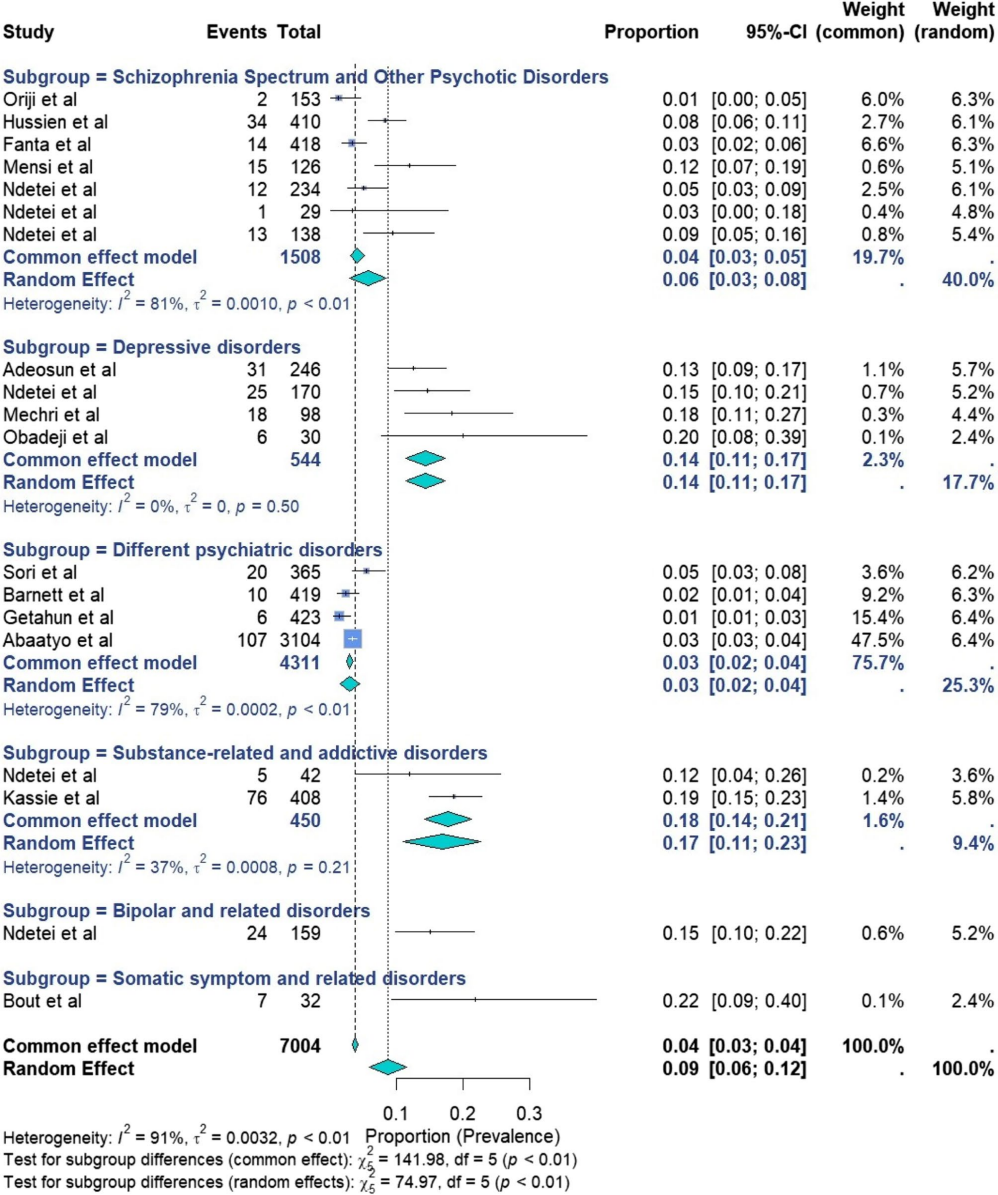
Forest plot for the pooled prevalence of recent suicide attempts

Nineteen studies assessing recent SA across various psychiatric disorders were included in the final meta-analysis. Some studies contributed data to multiple subgroups, as they reported on more than one psychiatric disorder type. The reported rates ranged from 1% in Nigeria [108] and Ethiopia [59] to 22% in Morocco [32] (Fig. 2). Owing to high heterogeneity, random-effects models were used. The pooled prevalence was 9% (95% CI: 6%−12%; I² = 91%, *p* < 0.01, *n* = 7004). Heterogeneity was confirmed with Cochran’s Q (Q = 192.23). Among the 19 studies, 4 focused on individuals with different psychiatric disorders, 7 focused on the schizophrenia spectrum and other psychotic disorders, 4 focused on depressive disorders, and 2 focused on substance-related and addictive disorders. The respective pooled prevalence rates were 3% (95% CI 2–4%) with (I^2^: 79%, *p* < 0.01), 6% (95% CI 3–8%) with (I^2^: 81%, *p* < 0.01), 14% (95% CI 11–17%) with (I^2^: 0%, *p* = 0.50), and 17% (95% CI 11–23%) with (I^2^: 37%, *p* = 0.21). Single studies revealed a prevalence of 15% (95% CI 10%−22%) for bipolar disorders and 22% (95% CI 9%−40%) for somatic symptom-related disorders (SSD). The highest weighted prevalences, reflecting each psychiatric disorder contribution to the overall burden, were observed in substance-related and addictive disorders (3.9%), schizophrenia spectrum and psychotic disorders (3.1%), and depressive disorders (2.6%).

**Fig. 3:**
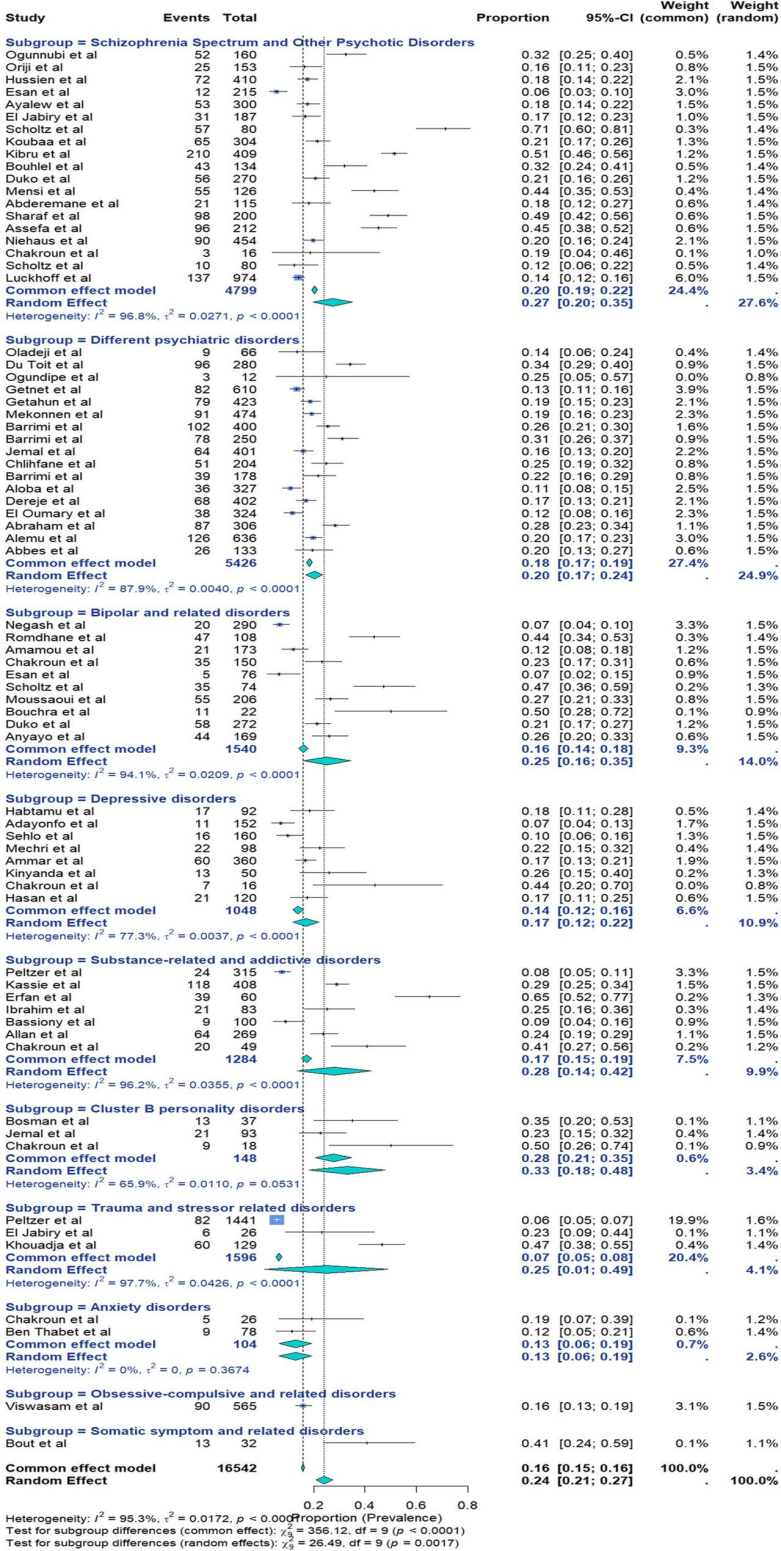
Forest plot for the pooled prevalence of lifetime suicide attempts

Seventy-one studies on lifetime suicide attempts were included. Some studies contributed data to multiple subgroups. The reported data ranged from 6% in South Africa and Nigeria [72, 96] to 71% in South Africa [81] (Fig. 3).

The random-effects model results were selected due to high heterogeneity. The pooled prevalence was 24% (95% CI: 21%−27%; I²=95.3%; *p* < 0.01; *n* = 16,542), with a Cochran’s Q = 1483.29. Given the high heterogeneity observed from the variance between the included studies, a subgroup analysis based on the DSM-defined psychiatric disorder classification was employed. Among the 71 studies, 19 addressed schizophrenia spectrum disorders and other psychotic disorders, 17 focused on different psychiatric disorders, 10 focused on bipolar and related disorders, 8 focused on depressive disorders, 7 focused on substance-related and addictive disorders, 3 focused on cluster B personality disorders, 3 focused on trauma- and stressor-related disorders, and 2 focused on anxiety disorders. The respective pooled prevalence of lifetime suicide attempts was 27% (95% CI 20–35%) with (I^2^ : 96.8%, *p* < 0.01), 20% (95% CI 17–24%) with (I^2^ : 87.9%, *p* < 0.01), 25% (95% CI 16–35%) with (I^2^ : 94.1%, *p* < 0.01), 17% (95% CI 12–22%) with (I^2^ : 77.3%, *p* < 0.01), 28% (95% CI 14–42%) with (I^2^ : 96.2%, *p* < 0.01), 33% (95% CI 18–48%) with (I^2^ : 65.9%, *p* = 0.05), 25% (95% CI 1–49%) with (I^2^ : 97.7%, *p* < 0.01), and 13% (95% CI 6–19%) with (I^2^ : 0%, *p* = 0.37). The remaining 2 studies focused on obsessive-compulsive and related disorders and somatic symptoms and related disorders, with pooled prevalences of 16% (95% CI 13–19%) and 41% (95% CI 24–59%), respectively. In a case-control study of patients with psychogenic nonepileptic seizures (PNES) [56], the pooled prevalence of lifetime suicide attempts for somatic symptoms and related disorders was 20%. The highest weighted prevalences, reflecting each psychiatric disorder contribution to the overall burden, were observed in schizophrenia spectrum and psychotic disorders (11.7%), trauma- and stressor-related disorders (3.6%), bipolar and related disorders (3.5%), and substance-related and addictive disorders (3.2%).

**Fig. 4:**
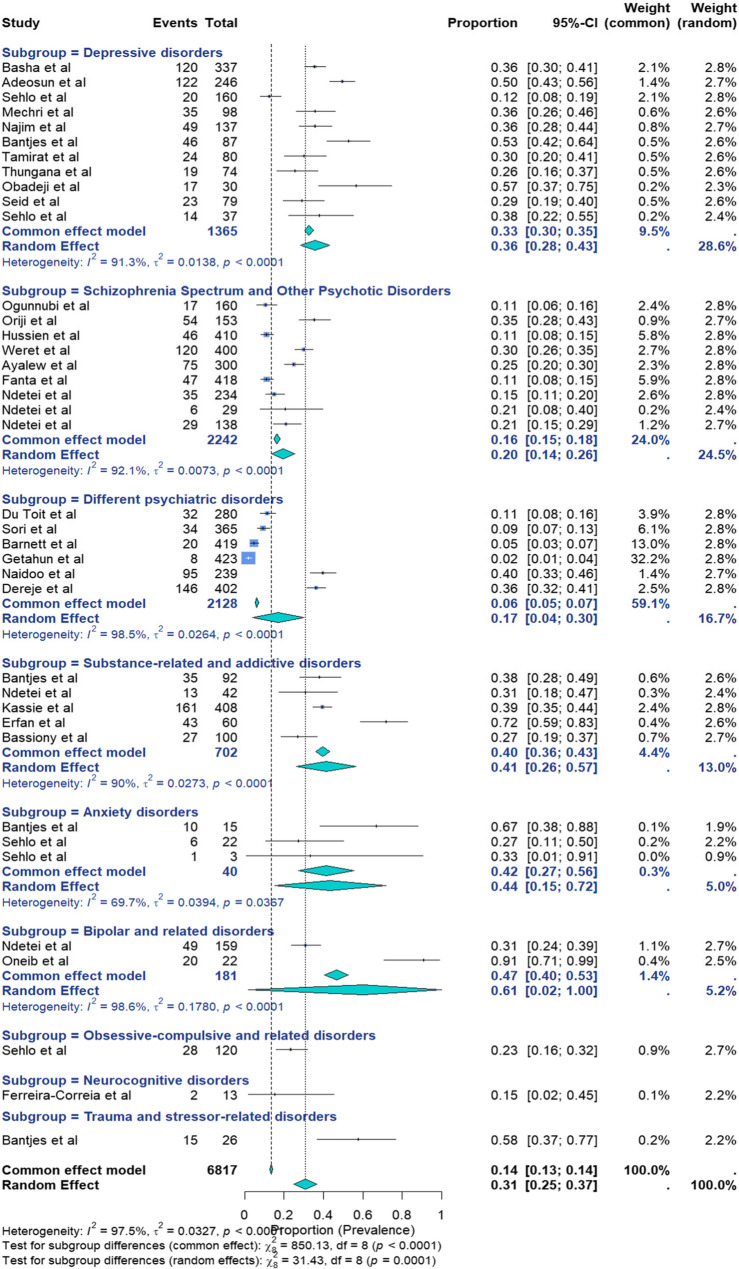
Forest plot for the pooled prevalence of recent suicidal ideations

Thirty-nine studies evaluating recent SI were included. Some studies contributed data to multiple subgroups. The prevalence ranged from 2% in Ethiopia [59] to 72% in Egypt [104] (Fig. 4). Given the high degree of heterogeneity, the results from a random-effects model were selected. The pooled prevalence was estimated at 31% (95% CI: 25%−37%; I² = 97%; *p* < 0.01; *n* = 6817). Heterogeneity was assessed using Cochran’s Q statistic, yielding Q = 1509.04, indicating substantial heterogeneity among the studies included. Among the 39 studies, 11 focused on depressive disorders, 9 focused on schizophrenia spectrum disorders and other psychotic disorders, 6 focused on studies addressing different psychiatric disorders, 5 focused on substance-related and addictive disorders, 3 focused on anxiety disorders, and **2** focused on bipolar and related disorders. The pooled prevalence of recent suicidal ideations was 36% (95% CI 28–43%) with (I^2^ : 91%, *p* < 0.01), 20% (95% CI 14–26%) with (I^2^ : 92%, *p* < 0.01) *and a point prevalence of 17.7% (95% CI 6.7–28.7%; I*^*2*^: *95.61%*,*p* < 0.01,*n* = 965), 17% (95% CI 4–30%) with (I^2^ : 98%, *p* < 0.01), 41% (95% CI 26–57%) with (I^2^ : 90%, *p* < 0.01), 44% (95% CI 15–72%) with (I^2^ : 70%, *p* = 0.04) and 61% (95% CI 2−100%) with (I^2^ : 99%, *p* < 0.01). The remaining 3 studies focused on: neurocognitive disorders: One study reported a pooled prevalence of 15% (95% CI 2–45%). Another study on obsessive‒compulsive and related disorders reported a pooled prevalence of 23% (95% CI 16–32%), and one study on trauma- and stressor-related disorders reported a pooled prevalence of 58% (95% CI 37–77%). The highest weighted prevalences, reflecting each psychiatric disorder contribution to the overall burden, were observed in depressive disorders (10.2%) and schizophrenia spectrum and psychotic disorders (9.3%).

**Fig. 5:**
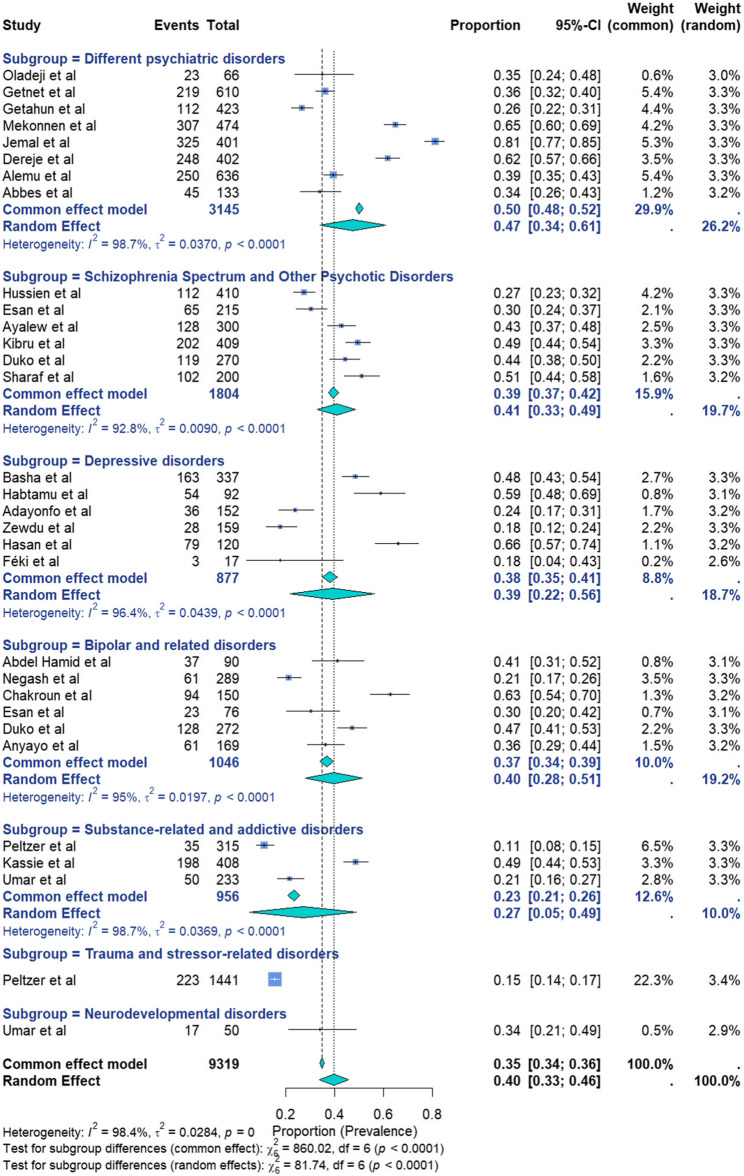
Forest plot for the pooled prevalence of lifetime suicidal ideations

Thirty-one studies assessing lifetime SI were included. Some studies contributed data to multiple subgroups. The reported prevalence rates ranged from 11% in South Africa [72] to 81% in Ethiopia [92] (Fig. 5). Owing to high heterogeneity, a random-effects model was applied, yielding a pooled prevalence of 40% (95% CI: 33%−46%; I²=98.4%; *p* = 0; *n* = 9319). Cochran’s Q statistic (Q = 1866.25) confirmed substantial heterogeneity. Among the 31 studies, 8 focused on different psychiatric disorders, 6 focused on the schizophrenia spectrum and other psychotic disorders, 6 focused on depressive disorders, 6 focused on bipolar and related disorders, and 3 focused on substance-related and addictive disorders. The respective pooled prevalence of lifetime suicidal ideations was 47% (95% CI 34–61%) with (I^2^ : 98.7%, *p* < 0.01), 41% (95% CI 33–49%) with (I^2^ : 92.8%, *p* < 0.01), 39% (95% CI 22–56%) with (I^2^ : 96.4%, *p* < 0.01), 40% (95% CI 28–51%) with (I^2^ : 95%, *p* < 0.01), and 27% (95% CI 5–49%) with (I^2^ : 98.7%, *p* < 0.01). The remaining 2 studies focused on neurodevelopmental disorders: one study had a pooled prevalence of 34% (95% CI 21%−49%), and one study on trauma- and stressor-related disorders had a pooled prevalence of 15% (95% CI 14–17%).The highest weighted prevalences, reflecting each psychiatric disorder contribution to the overall burden, were observed in schizophrenia spectrum and psychotic disorders (12.7%), bipolar disorders (8.0%), and depressive disorders (5.9%).

After studies were removed one by one in the sensitivity analyses, no outlying studies that could significantly change the primary results were found (Supplementary Material SM.3). Funnel plots displayed asymmetries for all outcomes except for lifetime suicidal ideation. Egger’s tests indicated significant publication bias for three outcomes, with a tendency for studies reporting larger effects (or more extreme results) to be more likely published: lifetime prevalence of suicide attempts (z = 5.012, *P* < 0.001), recent prevalence of suicide attempts (z = 4.487, *P* < 0.001), and recent prevalence of suicidal ideation (z = 3.117, *P* = 0.002). However, publication bias was not significant for the lifetime prevalence of suicidal ideation (z = −0.231, *P* = 0.818). The negative z score suggests that smaller studies with smaller effects might not be published, but this result is not statistically significant (Supplementary Material SM.5).

## Discussion

To the best of our knowledge, this is the first systematic review and meta-analysis to investigate the prevalence of STBs among psychiatric populations in Africa. The findings of 88 studies conducted between 2000 and 2023 (52 on ideation, 74 on attempts) provide valuable insights for public health policies and mental health interventions across the continent. In this meta-analysis, the pooled prevalence rates of lifetime and point suicidal ideation among individuals with schizophrenia were 41% and 17.7%, respectively. This finding differs from global estimates, where the prevalence of lifetime and point SI among people with schizophrenia was 34.5% and 29.9%, respectively, and in China, it was 19.22% and 18.06%, respectively [117, 118]. In a cohort study in the USA, adult Medicare patients with schizophrenia were found to have a lifetime SI of 9.7% [119]. Overall, the cross-national lifetime prevalence of SI among general population in 17 countries was found to be 9.2% [120], and in a meta-analysis performed on the European general population, the pooled point and lifetime prevalence of SI were found to be 5.28% and 9.08%, respectively [121].

The variability in prevalence likely reflects differences in methodologies, sample characteristics, and contextual factors. High heterogeneity (I² >90%) indicates underlying variability, possibly influenced by sample sizes, differing definitions of STBs, and timeframe variations. Religious or social beliefs may inhibit disclosure of recent suicidal ideation but not necessarily lifetime thoughts that could be more readily disclosed during retrospective questioning. Comparisons between psychiatric disorder subgroups must account for factors such as comorbid conditions, treatment adherence, disorder onset and severity, demographic variables, hospitalization history, substance use, social support, family history of psychiatric illness, and study setting. On a country-by-country level, the lifetime prevalence rate in China was notably lower than our pooled estimate. This may reflect a cultural environment where mental illness carries significant stigma, often leading to concealment of symptoms and underreporting, with notable differences between rural and urban settings, gender, and age [122]. This may also be explained by the profound social changes, including economic growth, increased urbanization – as suicide risk was highly associated with rural areas – an aging populace, and improved management of lethal substances that China has undergone which contributed to a considerable reduction in overall suicide mortality rates over recent decades, which may explain the lower rates in suicidal thoughts [123, 124]. China has also implemented mental health services within the Three-Tier Network of Health Care model, with social and educational programs for suicide prevention among rural residents [125]. Shifts in demographic and economic factors have also had clinical impacts on vulnerable groups, including individuals with disabilities such as psychiatric disorders–schizophrenia among them. This is reflected in the decline in treatment-naïve schizophrenia patients from 30.6% in 1994 to 20.4% in 2008, a change associated with improved access to care, higher education levels, increased availability of mental health professionals, greater financial resources, reduced treatment burden, and stronger social support systems [126, 127]. On a different level, traditional family structures and Confucian values also emphasize social harmony and familial duty, which could make these cultural frameworks discourage individuals from seeking help outside the family, making family conflict or shame often go unreported and unaddressed, elevating suicide risk [128]. At the same time, these cultural norms have been associated with reduced suicide risk among men, as shown in previous research [129]. In the United States, the lifetime prevalence of suicidal ideation among adult Medicare beneficiaries with schizophrenia was reported at just 9.7% -a notably low rate compared to estimates from Africa- which may be partially attributed to the demographic characteristics of the Medicare population, over 90% of whom are older adults aged 65 and above [130]. It was found that the relatively low rates of suicidal ideation observed among older adult Medicare patients with schizophrenia may be explained by several factors : as patients age, some experience functional recovery, symptom stabilization, or gradual improvement in general psychopathology, leading to fewer psychiatric hospitalizations and emergency visits related to suicidality [131]. Additionally, comorbid substance use disorders, which increase suicide risk, tend to be less common in older adults, though depression remains prevalent [132]. A healthy survivor effect also likely plays a role: those who reach older age may represent a subset who have survived earlier high-risk periods for suicide, possibly due to better coping strategies or protective health behaviors [133]. Moreover, individuals most vulnerable to suicide may have already died earlier, leaving a population with lower ongoing suicide risk [131]. Additionally, although individualism and weaker social ties in the U.S. may heighten risk in the general population, Medicare patients often benefit from more stable access to mental health care, which can foster better social support and connectivity, helping to mitigate that risk [134, 135]. Globally, Africa has consistently reported some of the highest suicide mortality rates, exceeding global averages [13]. Among vulnerable populations–such as individuals with schizophrenia–limited access to mental health services and poor treatment availability further compound the risk and may explain differences in lifetime SI among individuals with schizophrenia [136]. The lifetime prevalence of SI in African individuals with schizophrenia is 4.45 times higher than that observed in the general population worldwide –which is consistent with evidence from the literature– and it also exceeds the global average reported for individuals with schizophrenia [119, 137]. The contributing factors include under-resourced healthcare systems in terms of skilled personnel, facilities, and funding, leading to limited access to quality mental health care, which can result in inadequate treatment for individuals with schizophrenia, thereby exacerbating their condition and increasing the risk of SI. Mental health issues in Africa are frequently accompanied by significant social stigma. Individuals with schizophrenia may face discrimination and marginalization, leading to feelings of isolation and hopelessness, which can contribute to SI. A review indicated that 29% of individuals with mental illness in Africa experience internalized stigma (25.08% for individuals with schizophrenia), which is lower than rates reported elsewhere (56%) and is influenced by marital status, STBs, unemployment, poor social support, and literacy levels [138, 139]. Likewise, the pooled prevalence of lifetime SA among psychiatric individuals was 24%, notably higher than rates reported in African (9.9%) and European general populations (2.88%) [15, 121]. However, general samples include undiagnosed or untreated psychiatric individuals, complicating direct comparisons. Psychiatric disorders increase the risk of STBs through stigma, social withdrawal, hopelessness, substance abuse, financial instability, relationship stress, social injustice, and illiteracy, which may be influenced by environmental, genetic, or disorder-specific factors [140]. According to a 50 years of research meta-analysis, risk factors for SA include, but are not limited to : experiencing and being exposed to self-injurious thoughts and behaviors; general psychopathology; internalizing or externalizing psychopathology; family history of psychopathology and social factors. Predictors of SA included: prior non-suicidal self-injurious behavior or SA, personality disorders of any kind and prior psychiatric hospitalization [4].

Subsequently, the likelihood of occurrence and transition between SI to SA is higher among individuals with psychiatric disorders, which suggests the presence of mediators making these individuals more vulnerable to STBs. On the one hand, social or environmental factors, such as childhood sexual abuse, parenting behavior, parental loss, and certain stressful life events, are causally linked to an increased risk of psychiatric disorders, but their effects tend to be nonspecific [141]. Coping and emotion regulation mediate and moderate the association between stress exposure and psychopathology [142]. Another critical intermediary highlighted in the literature is represented by genetic predisposition [141]. Genes play a pivotal role in modulating an individual’s sensitivity to, control over, and exposure to environmental influences, which represent complex interactions that shape susceptibility to suicide. The evidence indicates that the latest estimate for the heritability of SA/completion is 43% [143]. Family genetic studies suggest that STBs are transmitted independent of psychopathology [144, 145]. This finding may contribute to explaining how individuals with no psychopathology may engage in suicidal behaviors and how the majority of patients with a psychiatric disorder do not attempt or die by suicide [146–148]. This further supports the suggestions of some studies in which suicidal behavior may constitute a distinct clinical syndrome coexisting with other forms of psychopathology [149]. Therefore, research must further explore proximal factors influencing suicide among both psychiatric and non-psychiatric individuals.

Countries studied vary significantly in terms of legal framework, access to mental healthcare, gender equality, culture, and religious context [150]. Drawing conclusions for psychiatric populations adds further complexity. In Ethiopia, where the dominant Ethiopian Orthodox Church is supplemented by a significant Muslim minority, religious and traditional beliefs strongly influence social attitudes [151]; mental health services are scarce, with only 0.1088 psychiatrists per 100,000 people, compared with the WHO recommendation of 0.898 per 100,000 [152, 153]. Studies from capital areas indicate rates of approximately 5.40 suicides per 100,000 people as of 2019 [154]. In Sudan and several North African countries, suicide is considered a religious transgression, which likely affects the accuracy of reported data [155–161]; this is reflected in official rates that appear low (often 3–7.5 per 100,000) but are widely believed to be underreported due to both stigma and concerns about potential legal or social consequences [162–168]. Nigeria’s religious diversity: With a predominantly Muslim north and a largely Christian south, regional variations are evident [169]. Although the official figures may indicate very low suicide rates (3.5 per 100,000 in 2019), these numbers are likely distorted by significant underreporting–partly because Nigerian law still criminalizes attempted suicide under Sect. 327 of its Criminal Code and 231 of its Penal Code [170–172]. Similarly, Uganda, Kenya, and Malawi–Christian-majority countries– criminalize suicide attempts [172–175]. In Uganda and Malawi (Sect. 229), survivors face prosecution [176–178]. Kenya’s Sect. 226 punished attempted suicide until a 2025 court ruling declared the law unconstitutional, potentially shifting public attitudes [179]. Despite the legal pressures in these countries, reported suicide rates remain on the lower side (approximately 4–7 per 100,000): Uganda, Malawi, and Kenya reported crude suicide rates of 4.6, 5.4 and 6.1 per 100,000, respectively, by 2019, although underreporting remains a concern [180–183]. South Africa, while having better urban mental health services, faces rural shortages in trained professionals and community-based support [184]. Tunisia and Egypt, benefiting from more developed healthcare systems in major urban centers, still struggle with underfunding and pervasive stigma, which limits the effective treatment of psychiatric disorders [185–188]. Nigeria, Uganda, Kenya, and Malawi face severe shortages of mental health specialists and large treatment gaps [189–192]. Ultimately, while South Africa sometimes reports higher suicide rates (23.5 per 100,000 in 2019–ranking third worst on the continent compared to other African nations) [193, 194], the very low official rates in countries such as Nigeria are understood to reflect legal sanctions and social stigma more than an accurate epidemiological picture [195, 196]. Among the 44 countries lacking STB studies among psychiatric patients in our systematic review, Lesotho and Eswatini are particularly notable, ranking first and third worldwide in crude suicide rates at 87.5 and 40.9 per 100,000, respectively. This further emphasizes the lack of research among the studied population, as reflected by these heightened rates [197–199]. The interplay of strong religious teachings, legal frameworks ranging from criminalization to recent moves toward decriminalization, and the uneven availability of mental health services means that, in these countries, the experience of STBs is deeply contextual [150, 200].

Subgroup analysis revealed that individuals with cluster B personality disorders had the highest lifetime rate of SA (33%), followed by those with substance use disorders (28%), schizophrenia spectrum and related disorders (27%), trauma and stressor-related disorders (25%), bipolar disorders (25%), SSD-related disorders (20%) –representing the pooled prevalence of all studies included in the systematic review on SSD, while the 41% prevalence reported in the single cross-sectional study remains insufficient to be considered the highest pooled prevalence due to its very small sample size– and depressive disorders (17%). Obsessive-compulsive and related disorders (OCRDs, 16%) and anxiety disorders (13%) had comparatively lower rates. High lifetime SA rates are commonly linked to mood disorders, substance use disorders, psychoses, and personality disorders [201–203]. Extensive research on the relationship between STBs and SSD indicates that 13–67% of participants reported a prior SA [204], placing the prevalence of SSDs in Africa within this range. Conversely, some studies reported lifetime suicide rates of 6% among individuals with anxiety disorders, with SA ranging from minimal or uncertain intent to severe attempts or completed suicide [205]. The elevated rates in our study could be attributed to differences in patient characteristics such as psychiatric comorbidities, variations in the definitions of outcomes, treatment adherence, disorder age of onset, and/or differences in sample size, which were relatively small in our study. It is important to recognize that small sample sizes in epidemiological surveys can lead to unstable findings, increasing the risk of selection bias [206].

Lifetime SA rates for OCRDs were previously reported to be 13.7% for OCD, alongside other obsessive‒compulsive and related disorders [207]. Our meta-analysis included only one study, so conclusions regarding OCRDs in Africa should be drawn with caution. However, we may consider that the high rate of OCD in the African sample, compared with other geographic regions, might be influenced by the co-occurrence of psychiatric disorders increasing the risk of STBs [208, 209]. Overall, determining which primary psychiatric disorders have the highest SA rates is challenging due to factors such as psychiatric comorbidities and other methodological barriers. Additionally, including suicidal behavior as a diagnostic criterion for BPD or MDD can introduce bias in studies assessing SA rates across different psychiatric disorders. This inherent criterion may lead to an apparent overrepresentation of SA among these individuals, potentially inflating the perceived prevalence of such behaviors compared with others where STBs are not a diagnostic feature and could be underreported. In our meta-analysis, no studies on cluster A and C personality disorders were retrieved, suggesting that suicide attempts may be less frequent in these groups, underexplored, or potentially underdiagnosed compared to cluster B disorders. The rates of substance abuse and related disorders may be influenced by the specific substances involved, whether through individual use or polysubstance use. In our study, alcohol, prescribed opioids, cannabis, and pregabalin were the most commonly used substances. Research has suggested that addiction to alcohol, pain relievers, marijuana, and cocaine might serve as stable and reliable predictors of STBs, which is the focus of our findings on these substances [210].

Subgroup analyses of SI showed higher recent SI than lifetime SI in bipolar (61% vs. 40%) and trauma-related disorders (58% vs. 15%), and slightly greater recent SI in substance-related disorders (41% vs. 27%). Depressive disorders showed consistent rates (39% lifetime vs. 36% recent). Schizophrenia individuals had a longer lifetime SI (41%) than recent SI (20%). The lifetime SI prevalence in neurodevelopmental disorders was 34%, whereas recent SI rates were 44% in anxiety disorders and 23% in OCRDs. An Italian study reported similar intake rates: bipolar disorder (42.4%), OCRDs (36.7%), depressive disorders (36.1%, and 36.6% for MDD), psychotic disorders (30%), SUDs (25.4%) and anxiety disorders (8.7%) [211]. Bipolar disorder has the highest SI rates among psychiatric conditions, nearly 20–30 times higher than that of the general population, with approximately 79% experiencing SI during the depression phase of the disorder [8]. Globally, lifetime SI prevalence in MDD is about 40.3% [212]. Depression and hopelessness are recognized as major risk factors for SI and are routinely incorporated into structured suicide risk assessments [17, 213]. A recent study on ADHD reported an SI prevalence of 25.1% in adolescents with ADHD compared with 10.3% in controls, with ADHD being a strong STB predictor (OR = 2.18) [214]. In our meta-analysis, we focused on SI among adults with ADHD due to limited available data, noting that high rates may reflect small sample sizes, psychiatric comorbidities, or the direct effects of ADHD. This impact may be explained by well-documented challenges associated with ADHD in adulthood, such as higher rates of college dropout, poorer job performance, difficulty maintaining employment, and lower earnings compared to peers of similar intellectual ability [215, 216]. Posttraumatic stress disorder (PTSD) has also been consistently linked with SI, with studies mainly focusing on military personnel and veterans, in which lifetime rates of SI ranged from 17.39% to 71.89% [217, 218]. In our meta-analysis, PTSD was the only trauma-related disorder assessed over a lifetime timeframe within a tuberculosis-diagnosed population, complicating interpretation due to the interaction between PTSD, STBs, and tuberculosis [219]. In the recent timeframe, PTSD and acute stress disorder were examined in a small group seeking HIV testing, with potential comorbidities such as MDD and AUD influencing the prevalence. Anxiety disorders were assessed in small samples, mostly focusing on GAD and panic disorder, which are often comorbid with other psychiatric or physical illnesses. Few studies have adequately addressed the combined mental and physical health burden on suicide risk. Nevertheless, anxiety disorders are consistently correlated with increased STB frequency, consistent with prior findings [220].

This systematic review and meta-analysis has several strengths, including the inclusion of a large number of studies from various countries, a substantial overall sample size, the application of advanced analytical methods, and the diversity of subgroup diagnoses of psychiatric disorders, enabling an assessment of outcomes across a wide spectrum of disorders. However, our study results should be considered in light of several limitations. Geographically, only 10 out of 54 African countries were represented due to insufficient data from the remaining countries, limiting the generalizability of the findings to the entire continent. Not all psychiatric disorders were covered–for example, neurodevelopmental disorders excluded individuals with intellectual disabilities and autism spectrum disorders. Some diagnoses, such as disruptive, impulse-control, and conduct disorders; feeding and eating disorders; sexual dysfunctions; and sleep‒wake disorders, were not reported because of the absence of available data. Additionally, the timeframes of STBs have been inconsistently reported, requiring analysis to rely on conclusions derived from the text in some cases. In the “recent STBs” category, which spans from two weeks to one year prior, not all studies encompass the full timeframe. For example, some individuals may not have experienced STBs in the past two weeks (at the time of the study) but may have experienced them within the past year. As a result, the estimated prevalence may be underestimated. Additionally, many included studies had small sample sizes, which may affect the precision of the pooled estimates. Non-probability sampling was used in most studies, limiting the generalizability of the findings. Also, the lack of standardized diagnostic and assessment tools likely led to misclassification of psychiatric conditions and inaccurate STB reporting. Data on lifetime psychopathology were also not always available, preventing an assessment of the long-term impact of psychiatric disorders on STBs. Information on current treatments has not been consistently reported, making it difficult to account for the potential influence of some treatment regimens on STBs. Heterogeneity in the meta-analysis of epidemiological studies could not be fully avoided, despite subgroup analyses. Furthermore, data on STBs were retrospectively collected in some studies, increasing the risk of recall bias, and some of these outcomes were self-reported, which might impact the overall rates in terms of the data collected (SBQ-R, BDI-II, CDI, EPDS-10, etc.). Finally, only published studies were included because of limited access to unpublished data. This makes data underestimation a major limitation in the present research, as the meta-analyzed prevalence of STBs is drawn from earlier studies that likely relied on inaccurate or incomplete evaluations. Therefore, there is a pressing need for future research to prioritize the development of more rigorous and comprehensive data collection systems.

In summary, STBs among individuals with psychiatric disorders pose a significant public health concern in Africa. This review reveals considerable variation in STB prevalence by diagnosis and region: recent and lifetime suicide attempts accounted for 9% and 24%, respectively, and suicidal ideation accounted for 31% and 40%, respectively. These findings underscore the urgent need for targeted mental health interventions, particularly for mood, schizophrenia, and substance-related disorders. By refining global estimates, this study contributes to both regional policy development and the broader understanding of suicide-related outcomes in high-risk populations.

## Supplementary Information

Supplementary Material 1.

## Data Availability

All data extracted and analyzed during this study are included in this published article.

## Abbreviations

SA: Suicide attempt
SI: Suicidal ideation
CI: Confidence interval
STBs: Suicidal thoughts and behaviors
