# Supplementary material for: Prevalence of suicidal thoughts and behaviors among individuals with mental disorders in Africa: a systematic review and meta-analysis of the past 25 years
Source: BMC Psychiatry. 2026 Feb 12;26:158. doi: 10.1186/s12888-025-07453-4 (PMC12895970; doi:10.1186/s12888-025-07453-4)
Supplement: Supplementary file 1 — Supplementary Material 1. [file 12888_2025_7453_MOESM1_ESM.docx]

**SM Table 1 : Search strategy :**

| Database/ search date | | Search strategy : |
| --- | --- | --- |
| Pubmed | (((((((((((((suicid) OR (suicide ideation)) OR (suicidal ideation)) OR (suicide attempt)) OR (attempted suicide)) OR (suicidality)) OR (suicidal behavior)) OR (suicidal thoughts)) OR (suicide)) **AND** (((((((((((((((((((((((((((((((((((((((((((((((((((((((((((((((((((((((((((((((((((((((((((((((((((((((((((((((((((((((((((((((((((((((((((((((((((((((((((((((((((((((((((((((((((((((((((((((((((((((((((((Morocco[Title/Abstract]) OR (Tunisia[Title/Abstract])) OR (algeria[Title/Abstract])) OR (Angola[Title/Abstract])) OR (Benin[Title/Abstract])) OR (Botswana[Title/Abstract])) OR (Burkina Faso[Title/Abstract])) OR (Burundi[Title/Abstract])) OR (Cabo Verde[Title/Abstract])) OR (Cameroon[Title/Abstract])) OR (Central African Republic[Title/Abstract])) OR (Chad[Title/Abstract])) OR (Comoros[Title/Abstract])) OR (Democratic Republic of the Congo[Title/Abstract])) OR (Djibouti[Title/Abstract])) OR (Egypt[Title/Abstract])) OR (Equatorial Guinea[Title/Abstract])) OR (Eritrea[Title/Abstract])) OR (Eswatini[Title/Abstract])) OR (Ethiopia[Title/Abstract])) OR (Gabon[Title/Abstract])) OR (Gambia[Title/Abstract])) OR (Ghana[Title/Abstract])) OR (Guinea[Title/Abstract])) OR (Guinea-Bissau[Title/Abstract])) OR (Ivory Coast[Title/Abstract])) OR (Kenya[Title/Abstract])) OR (Lesotho[Title/Abstract])) OR (Liberia[Title/Abstract])) OR (Libya[Title/Abstract])) OR (Madagascar[Title/Abstract])) OR (Malawi[Title/Abstract])) OR (Mali[Title/Abstract])) OR (Mauritania[Title/Abstract])) OR (Mauritius[Title/Abstract])) OR (Mozambique[Title/Abstract])) OR (Namibia[Title/Abstract])) OR (Niger[Title/Abstract])) OR (Nigeria[Title/Abstract])) OR (Republic of the Congo[Title/Abstract])) OR (Rwanda[Title/Abstract])) OR (Sao Tome and principe[Title/Abstract])) OR (Senegal[Title/Abstract])) OR (Seychelles[Title/Abstract])) OR (Sierra Leone[Title/Abstract])) OR (Somalia[Title/Abstract])) OR (South Africa[Title/Abstract])) OR (South Sudan[Title/Abstract])) OR (Sudan[Title/Abstract])) OR (Tanzania[Title/Abstract])) OR (Togo[Title/Abstract])) OR (Uganda[Title/Abstract])) OR (Zambia[Title/Abstract])) OR (Zimbabwe[Title/Abstract])) OR (algeria[MeSH Terms])) OR (angola[MeSH Terms])) OR (benin[MeSH Terms])) OR (republic of benin[MeSH Terms])) OR (botswana[MeSH Terms])) OR (burkina faso[MeSH Terms])) OR (burkina fasso[MeSH Terms])) OR (burundi[MeSH Terms])) OR (republic of burundi[MeSH Terms])) OR (cape verde[MeSH Terms])) OR (cameroon[MeSH Terms])) OR (united republic of cameroon[MeSH Terms])) OR (central african republic[MeSH Terms])) OR (chad[MeSH Terms])) OR (comoros[MeSH Terms])) OR (democratic republic of the congo[MeSH Terms])) OR (congo[MeSH Terms])) OR (djibouti[MeSH Terms])) OR (republic of djibouti[MeSH Terms])) OR (arab republic of egypt[MeSH Terms])) OR (egypt[MeSH Terms])) OR (republic of equatorial guinea[MeSH Terms])) OR (equatorial guinea[MeSH Terms])) OR (eritrea[MeSH Terms])) OR (ethiopia[MeSH Terms])) OR (federal democratic republic of ethiopia[MeSH Terms])) OR (gabon[MeSH Terms])) OR (gabonese republic[MeSH Terms])) OR (gambia[MeSH Terms])) OR (republic of the gambia[MeSH Terms])) OR (ghana[MeSH Terms])) OR (republic of ghana[MeSH Terms])) OR (republic of guinea[MeSH Terms])) OR (republic of guinea bissau[MeSH Terms])) OR (ivory coast[MeSH Terms])) OR (kenya[MeSH Terms])) OR (republic of kenya[MeSH Terms])) OR (lesotho[MeSH Terms])) OR (kingdom of lesotho[MeSH Terms])) OR (liberia[MeSH Terms])) OR (republic of liberia[MeSH Terms])) OR (libya[MeSH Terms])) OR (madagascar[MeSH Terms])) OR (malawi[MeSH Terms])) OR (republic of malawi[MeSH Terms])) OR (republic of mali[MeSH Terms])) OR (mauritania[MeSH Terms])) OR (mauritius[MeSH Terms])) OR (morocco[MeSH Terms])) OR (republic of mozambique[MeSH Terms])) OR (mozambique[MeSH Terms])) OR (namibia[MeSH Terms])) OR (republic of namibia[MeSH Terms])) OR (niger[MeSH Terms])) OR (republic of niger[MeSH Terms])) OR (federal republic of nigeria[MeSH Terms])) OR (nigeria[MeSH Terms])) OR (republic of the congo[MeSH Terms])) OR (republic of rwanda[MeSH Terms])) OR (rwanda[MeSH Terms])) OR (sao tome and principe[MeSH Terms])) OR (republic of senegal[MeSH Terms])) OR (senegal[MeSH Terms])) OR (seychelles[MeSH Terms])) OR (republic of sierra leone[MeSH Terms])) OR (sierra leone[MeSH Terms])) OR (somalia[MeSH Terms])) OR (republic of south africa[MeSH Terms])) OR (south africa[MeSH Terms])) OR (sudan[MeSH Terms])) OR (republic of the sudan[MeSH Terms])) OR (tanzania[MeSH Terms])) OR (united republic of tanzania[MeSH Terms])) OR (togo[MeSH Terms])) OR (togolese republic[MeSH Terms])) OR (tunisia[MeSH Terms])) OR (republic of uganda[MeSH Terms])) OR (uganda[MeSH Terms])) OR (republic of zambia[MeSH Terms])) OR (zambia[MeSH Terms])) OR (republic of zimbabwe[MeSH Terms])) OR (zimbabwe[MeSH Terms])) OR (algérie[Title/Abstract])) OR (bénin[Title/Abstract])) OR (cap-vert[Title/Abstract])) OR (République centrafricaine[Title/Abstract])) OR (Tchad[Title/Abstract])) OR (Guinée[Title/Abstract])) OR (Égypte[Title/Abstract])) OR (Éthiopie[Title/Abstract])) OR (Guinée-Bissau[Title/Abstract])) OR (Libéria[Title/Abstract])) OR (Nigéria[Title/Abstract])) OR (Sénégal[Title/Abstract])) OR (Soudan[Title/Abstract])) OR (Tanzanie[Title/Abstract])) OR (Tunisie[Title/Abstract])) OR (Zambie[Title/Abstract])) OR (Ouganda[Title/Abstract])) OR (Sao Tomé[Title/Abstract])) OR (Cameroun[Title/Abstract])) OR (Gambie[Title/Abstract])) OR (Libye[Title/Abstract])) OR (Maurice[Title/Abstract])) OR (Maroc[Title/Abstract])) OR (Namibie[Title/Abstract])) OR (Mauritanie[Title/Abstract])) OR (Somalie[Title/Abstract])) OR (Afrique du Sud[Title/Abstract])) OR (Dahomey[Title/Abstract])) OR (Bechuanaland[Title/Abstract])) OR (Burkina fasso[Title/Abstract])) OR (Upper volta[Title/Abstract])) OR (Cape verde[Title/Abstract])) OR (Haute-volta[Title/Abstract])) OR (congo[Title/Abstract])) OR (swaziland[Title/Abstract])) OR (Cote d'Ivoire[Title/Abstract])) OR (Congo-brazzaville[Title/Abstract])) OR (Brazzaville[Title/Abstract])) OR (zaire[Title/Abstract])) OR (dahomey[MeSH Terms])) OR (bechuanaland[MeSH Terms])) OR (upper volta[MeSH Terms])) OR (swaziland[MeSH Terms])) OR (cote d ivoire[MeSH Terms])) OR (cote d'ivoire[MeSH Terms])) OR (congo brazzaville[MeSH Terms])) OR (zaire[MeSH Terms])) OR (africa[MeSH Terms])) OR (africa south of the sahara[MeSH Terms])) OR (africa, central[MeSH Terms])) OR (africa, eastern[MeSH Terms])) OR (africa, northern[MeSH Terms])) OR (southern africa[MeSH Terms])) OR (africa, western[MeSH Terms])) OR (maghreb[MeSH Terms])) OR (north africa[MeSH Terms])) OR (south west africa[MeSH Terms])) OR (africa[Title/Abstract])) OR (West africa[Title/Abstract])) OR (east africa[Title/Abstract])) OR (eastern africa[Title/Abstract])) OR (Southern africa[Title/Abstract])) OR (north africa[Title/Abstract])) OR (Maghreb[Title/Abstract])) OR (central africa[Title/Abstract])) OR (western africa[Title/Abstract])) OR (northern africa[Title/Abstract])) OR (south west africa[Title/Abstract])) OR (africa south of the sahara[Title/Abstract]))) **AND** ((((((((((((((((prevalence[MeSH Terms]) OR (prevalence studies[MeSH Terms])) OR (prevalence study[MeSH Terms])) OR (analyses, cross sectional[MeSH Terms])) OR (analysis, cross sectional[MeSH Terms])) OR (prevalence)) OR (cross-sectional)) OR (burden)) OR (magnitude)) OR (Scale)) OR (M.I.N.I.)) OR (International Neuropsychiatric Interview)) OR (Interview)) OR (interview, psychologic[MeSH Terms])) OR (interview, psychological[MeSH Terms])) OR (questionnaire))) **AND** (("2000/01/01"[Date - Publication] : "3000"[Date - Publication]))) **NOT** (("case reports"[Publication Type] OR "comment"[Publication Type] OR "editorial"[Publication Type] OR "historical article"[Publication Type] OR "letter"[Publication Type] OR "meta analysis"[Publication Type] OR "retracted publication"[Publication Type] OR "systematic review"[Publication Type] OR "retraction of publication"[Publication Type] OR "duplicate publication"[Publication Type]) OR (((((((((((((((((((((Meta-analysis[Title]) OR (Systematic review[Title])) OR (Global school-based health survey[Title])) OR (refugee[Title])) OR (migrant[Title])) OR (case report[Title])) OR (editorial[Title])) OR (letter[Title])) OR (comment[Title])) OR (editorial[Title])) OR (historical article[Title])) OR (case series[Title])) OR (book[Title])) OR (refugee[MeSH Terms])) OR (migrant[MeSH Terms])) OR (emigrant[MeSH Terms])) OR (emigrants[MeSH Terms])) OR (emigrants and immigrants[MeSH Terms])) OR (immigrants and emigrants[MeSH Terms])) OR (immigrant[MeSH Terms])) OR (immigrants[MeSH Terms])))) **AND** (((((((((((((((((((((((((((((((((((((((((((((((((((((((((((((((((((((((((((((((((((((((((((((((((((((((((((((((mood disorder) OR (Major Depressive Disorder)) OR (Dysthymic Disorder)) OR (Bipolar I Disorder)) OR (Bipolar II Disorder)) OR (Cyclothymic Disorder)) OR (Mood Episode)) OR (Major Depressive Episode)) OR (Manic Episode)) OR (Hypomanic Episode)) OR (Mixed Episode)) OR (Depressive Disorders)) OR (Bipolar Disorders)) OR (Anxiety disorder)) OR (Panic Attack)) OR (Agoraphobia)) OR (panic disorder)) OR (Specific Phobia)) OR (Social Phobia)) OR (phobia)) OR (Obsessive-Compulsive Disorder)) OR (Posttraumatic Stress Disorder)) OR (Acute Stress Disorder)) OR (Generalized Anxiety Disorder)) OR (Dissociative disorder)) OR (Dissociative Amnesia)) OR (Dissociative Fugue)) OR (Dissociative Identity Disorder)) OR (Depersonalization Disorder)) OR (Personality disorder)) OR (Paranoid Personality Disorder)) OR (Schizoid Personality Disorder)) OR (Dependent Personality Disorder)) OR (Obsessive-Compulsive Personality Disorder)) OR (Schizotypal Personality Disorder)) OR (Antisocial Personality Disorder)) OR (Borderline Personality Disorder)) OR (Histrionic Personality Disorder)) OR (Narcissistic Personality Disorder)) OR (Avoidant Personality Disorder)) OR (Schizoaffective Disorder)) OR (Delusional Disorder)) OR (Brief Psychotic Disorder)) OR (Shared Psychotic Disorder)) OR (psychotic disorder)) OR (Schizophrenia)) OR (Schizophreniform Disorder)) OR (substance use disorder)) OR (substance dependence)) OR (substance abuse)) OR (substance-induced disorders)) OR (substance-related disorder)) OR (mental illness)) OR (mental disorder)) OR (mental health condition)) OR (psychiatric disorder)) OR (Somatoform disorder)) OR (Somatization Disorder)) OR (Undifferentiated Somatoform Disorder)) OR (Conversion Disorder)) OR (Pain Disorder)) OR (Hypochondriasis)) OR (Body Dysmorphic Disorder)) OR (Factitious disorder)) OR (Sexual and gender identity disorder)) OR (Paraphilias)) OR (Gender Identity Disorders)) OR (sexual disorder)) OR (Sexual Dysfunction)) OR (Eating disorder)) OR (Anorexia Nervosa)) OR (Bulimia Nervosa)) OR (Sleep disorder)) OR (Dyssomnia)) OR (Parasomnia)) OR (Impulse-control disorder)) OR (Intermittent Explosive Disorder)) OR (Kleptomania)) OR (Pyromania)) OR (Pathological Gambling)) OR (Trichotillomania)) OR (Adjustment disorder)) OR (Cognitive disorder)) OR (Delirium)) OR (Dementia)) OR (Amnestic Disorder)) OR (Catatonic Disorder)) OR (Catatonic Disorder)) OR (Neurodevelopmental disorder)) OR (Mental Retardation)) OR (Learning Disorder)) OR (Motor Skills Disorder)) OR (Communication Disorder)) OR (Pervasive Developmental Disorder)) OR (Autistic Disorder)) OR (Rett's Disorder)) OR (Childhood Disintegrative Disorder)) OR (Asperger's Disorder)) OR (Attention-Deficit and Disruptive Behavior Disorder)) OR (Feeding and Eating Disorder)) OR (Pica)) OR (Rumination Disorder)) OR (Tic Disorder)) OR (Tourette's Disorder)) OR (Elimination Disorder)) OR (Encopresis)) OR (Enuresis)) OR (Separation Anxiety Disorder)) OR (Selective Mutism)) OR (Reactive Attachment Disorder)) OR (Stereotypic Movement Disorder)) | |
| scopus | ( TITLE-ABS-KEY ( morocc* OR tunisia OR algeria OR angola OR benin OR botswana OR "Burkina Faso" OR burundi OR "Cabo Verde" OR cameroon OR "Central African Republic" OR chad OR comoros OR "Democratic Republic of the Congo" OR djibouti OR egypt OR "Equatorial Guinea" OR eritrea OR eswatini OR ethiopia OR gabon OR gambia OR ghana OR guinea OR "Guinea-Bissau" OR "Ivory Coast" OR kenya OR lesotho OR liberia OR libya OR madagascar OR malawi OR mali OR mauritania OR mauritius OR mozambique OR namibia OR niger OR nigeria OR "Republic of the Congo" OR rwanda OR "Sao Tome and Principe" OR senegal OR seychelles OR "Sierra Leone" OR somalia OR "South Africa" OR "South Sudan" OR sudan OR tanzania OR togo OR uganda OR zambia OR zimbabwe OR dahomey OR bechuanaland OR "Burkina fasso" OR "Upper volta" OR "Cape verde" OR haute-volta OR congo OR swaziland OR "Côte d'Ivoire" OR congo-brazzaville OR brazzaville OR zaire OR africa OR "West africa" OR "east africa" OR "eastern africa" OR "Southern africa" OR "north africa" OR maghreb OR "central africa" OR "western africa" OR "northern africa" OR "south west africa" OR "africa south of the sahara" ) AND TITLE-ABS-KEY ( suicid* ) **AND** TITLE-ABS-KEY ( prevalence OR cross-sectional OR burden OR magnitude OR scale OR "International Neuropsychiatric Interview" OR interview OR questionnaire OR descriptive OR "comparative study" ) AND ALL ( disorder OR episode OR "Panic Attack" OR agoraphobia OR phobia OR dissociative OR schizophrenia OR "substance dependence" OR "substance abuse" OR "substance-induced disorder" OR "mental illness" OR "mental disorder" OR "mental health condition" OR "mental* ill*" OR hypochondriasis OR paraphilias OR "Sexual Dysfunction" OR "Anorexia Nervosa" OR "Bulimia Nervosa" OR dyssomnia OR parasomnia OR kleptomania OR pyromania OR "Pathological Gambling" OR trichotillomania OR delirium OR dementia OR "Mental Retardation" OR adhd OR pica OR encopresis OR enuresis OR "Selective Mutism" OR "intellectual* disab*" OR autism OR "Gender dysphoria" OR malingering OR mania OR psychosurgery ) ) **AND** PUBYEAR > 1999 **AND** PUBYEAR < 2025 **AND** **NOT** ( TITLE ( meta-analysis OR "Systematic review" OR refugee OR migrant OR "case report" OR "case series" ) OR TITLE-ABS-KEY ( "Global school-based health survey" OR "historical article" ) OR KEY ( meta-analysis OR "Systematic review" OR refugee OR migrant OR "case report" OR "case series" ) ) AND ( LIMIT-TO ( DOCTYPE , "ar" ) ) | |
| Web of science | suicid* (Topic) **AND** morocc* OR tunisia OR algeria OR angola OR benin OR botswana OR "Burkina Faso" OR burundi OR "Cabo Verde" OR cameroon OR "Central African Republic" OR chad OR comoros OR "Democratic Republic of the Congo" OR djibouti OR egypt OR "Equatorial Guinea" OR eritrea OR eswatini OR ethiopia OR gabon OR gambia OR ghana OR guinea OR "Guinea-Bissau" OR "Ivory Coast" OR kenya OR lesotho OR liberia OR libya OR madagascar OR malawi OR mali OR mauritania OR mauritius OR mozambique OR namibia OR niger OR nigeria OR "Republic of the Congo" OR rwanda OR "Sao Tome and Principe" OR senegal OR seychelles OR "Sierra Leone" OR somalia OR "South Africa" OR "South Sudan" OR sudan OR tanzania OR togo OR uganda OR zambia OR zimbabwe OR dahomey OR bechuanaland OR "Burkina fasso" OR "Upper volta" OR "Cape verde" OR "haute-volta" OR congo OR swaziland OR "Côte d'Ivoire" OR "congo-brazzaville" OR brazzaville OR zaire OR africa OR "West africa" OR "east africa" OR "eastern africa" OR "Southern africa" OR "north africa" OR maghreb OR "central africa" OR "western africa" OR "northern africa" OR "south west africa" OR "africa south of the Sahara" (Topic) **AND** prevalence OR cross-sectional OR burden OR magnitude OR scale OR "International Neuropsychiatric Interview" OR interview OR questionnaire OR "M.I.N.I." OR "comparative study" OR "comparative studies" OR "descriptive" (Topic) **NOT** meta-analysis OR "Systematic review" OR refugee OR migrant OR "case report" OR "case series" (Title) **NOT** "Global school-based health survey" OR "historical article" (Topic) NOT meta-analysis OR "Systematic review" OR refugee OR migrant OR "case report" OR "case series" (Author Keywords) | |

**SM Table 2 : Quality assessment**

| **Authors** | **Study design** | **Were the criteria for inclusion in the sample clearly defined?** | **Were the study subjects and the setting described in detail?** | **Was the exposure measured in a valid and reliable way?** | **Were objective, standard criteria used for measurement of the condition?** | **Were confounding factors identified?** | **Were strategies to deal with confounding factors stated?** | **Were the outcomes measured in a valid and reliable way?** | **Was appropriate statistical analysis used?** |
| --- | --- | --- | --- | --- | --- | --- | --- | --- | --- |
| **Allan et al** | Cross-sectional | Yes | No | Yes | Yes | Yes | Yes | No | Yes |
| **Ibrahim et al** | Cross-sectional | Yes | Yes | Yes | Yes | No | No | No | Yes |
| **Abderemane et al** | Cross-sectional | Yes | Yes | Yes | Yes | No | No | Unclear | Yes |
| **Bout et al** | Cross-sectional | Yes | No | Yes | Yes | Yes | Yes | Yes | Yes |
| **Obadeji et al** | Cross-sectional | Yes | Yes | Yes | Yes | No | No | Yes | Yes |
| **Abdel Hamid et al** | Cross-sectional | Yes | Yes | Yes | Yes | Yes | Yes | No | Yes |
| **Ferreira-Correia et al** | Cross-sectional | Yes | No | Yes | Yes | Yes | Yes | Yes | Yes |
| **Sharaf et al** | Cross-sectional | Yes | Yes | Yes | Yes | Yes | Yes | No | Yes |
| **Negash et al** | Cross-sectional | Yes | No | Yes | Yes | NA | NA | Unclear | Yes |
| **Mechri et al** | Cross-sectional | Yes | No | Yes | Yes | Yes | Yes | Unclear | Yes |
| **Getnet et al** | Cross-sectional | Yes | Yes | Yes | Unclear | Yes | Yes | Unclear | Yes |
| **Amamou et aI** | Cross-sectional | Yes | Yes | Yes | Yes | NA | NA | Unclear | Yes |
| **Duko et al** | Cross-sectional | Yes | Yes | Yes | Yes | NA | NA | Yes | Yes |
| **Oladeji et al** | Cross-sectional | Yes | Yes | Yes | Yes | Yes | Yes | Yes | Yes |
| **Kibru et al** | Cross-sectional | Yes | Yes | Unclear | Unclear | Yes | Yes | Yes | Yes |
| Bram et al | Cohort | Yes | Yes | Yes | Yes | NA | NA | Unclear | Yes |
| **Barnett et al** | Cross-sectional | Yes | Yes | Yes | Yes | NA | NA | Unclear | Yes |
| **Ndetei et al** | Cross-sectional | Yes | Yes | Yes | Yes | NA | NA | Yes | Yes |
| **Ndetei et al** | Cross-sectional | Yes | Yes | Yes | Yes | NA | NA | Yes | Yes |
| **Assefa et al** | Cross-sectional | Yes | No | Yes | Yes | Yes | Yes | Yes | Yes |
| **Niehaus et al** | Cross-sectional | Yes | No | Yes | Yes | Yes | Yes | Yes | Yes |
| **Du Toit et al** | Cross-sectional | Yes | Yes | Yes | Yes | Yes | Yes | Yes | Yes |
| **Basha et al** | Cross-sectional | Yes | Yes | Unclear | Unclear | Yes | Yes | Yes | Yes |
| **Adayonfo et al** | Cross-sectional | Yes | Yes | Yes | Yes | Yes | Yes | Yes | Yes |
| **Kinyanda et al** | Cross-sectional | Yes | Yes | Yes | Yes | Yes | Yes | Yes | Yes |
| **Romdhane et al** | Cross-sectional | Yes | Yes | Yes | Yes | Yes | Yes | Yes | Yes |
| **Kassie et al** | Cross-sectional | No | Yes | Unclear | Yes | Yes | Yes | Yes | Yes |
| **Ahmed et al** | Cohort | Yes | Yes | Yes | Yes | No | No | Unclear | Yes |
| **Getahun et al** | Cross-sectional | Yes | Yes | Yes | Yes | Yes | Yes | Yes | Yes |
| Hasan et al | Cross-sectional | Yes | Yes | Yes | Yes | Yes | Yes | Yes | Yes |
| **Najim et al** | Cross-sectional | Yes | Yes | Yes | Yes | NA | NA | Unclear | Yes |
| **Bosman et al** | Cross-sectional | No | No | Unclear | Unclear | Yes | Yes | Yes | Yes |
| **Koubaa et al** | Cross-sectional | Yes | Yes | Yes | Yes | Yes | Yes | Unclear | Yes |
| **Féki et al** | Cross-sectional | Yes | Yes | Unclear | Yes | NA | NA | Yes | Yes |
| **Adeosun et aI** | Cross-sectional | Yes | Yes | Yes | Yes | Yes | Yes | Unclear | Yes |
| Doufik et al | Cohort | Yes | Yes | Yes | Yes | NA | NA | Unclear | Yes |
| **Bantjes et al** | Cross-sectional | Yes | No | Yes | Yes | Yes | Yes | Yes | Yes |
| Dereje et al | Cross-sectional | Yes | Yes | Yes | Yes | Yes | Yes | Yes | Yes |
| Moussaoui et al | Cross-sectional | Yes | Yes | Yes | Yes | NA | NA | Unclear | Yes |
| Ben Thabet et al | Cross-sectional | Yes | Yes | Yes | Yes | Yes | Yes | Yes | Yes |
| **Abaatyo et al** | Cross-sectional | Yes | Yes | Yes | Yes | Yes | Yes | Unclear | Yes |
| **Peltzer et al** | Cross-sectional | Yes | Yes | Yes | Yes | Yes | Yes | Unclear | Yes |
| **Habtamu et al** | Cross-sectional | Yes | Yes | Yes | Yes | Yes | Yes | Yes | Yes |
| **Tamirat et al** | Cross-sectional | Yes | Yes | Yes | Yes | Yes | Yes | Yes | Yes |
| **Viswasam et al** | Cross-sectional | No | Yes | Yes | Yes | No | No | Unclear | Yes |
| **Sori et al** | Cross-sectional | Yes | Yes | Unclear | Yes | Yes | Yes | Yes | Yes |
| **Zewdu et al** | Cross-sectional | Yes | Yes | Yes | Yes | Yes | Yes | Yes | Yes |
| **Anyayo et al** | Cross-sectional | Yes | Yes | Unclear | Unclear | Yes | Yes | Unclear | Yes |
| **Chakroun et aI** | Cross-sectional | Yes | Yes | Yes | Yes | Yes | Yes | Unclear | Yes |
| **Luckhoff et al** | Cross-sectional | Yes | No | Yes | Yes | Yes | Yes | Yes | Yes |
| **Scholtz et al** | Cross-sectional | Yes | No | Yes | Yes | Yes | Yes | Yes | Yes |
| **Bassiony et al** | Cross-sectional | Yes | Yes | Yes | Yes | Yes | Yes | No | Yes |
| **Mekonnen et al** | Cross-sectional | Yes | Yes | Yes | Yes | NA | NA | Unclear | Yes |
| **Ammar et al** | Cross-sectional | Yes | Yes | Yes | Yes | Yes | Yes | Unclear | Yes |
| **Sehlo et al** | Cross-sectional | Yes | Yes | Yes | Yes | Yes | Yes | Yes | Yes |
| **Sehlo et al** | Cross-sectional | Yes | Yes | Yes | Yes | Yes | Yes | Yes | Yes |
| **Ayalew et al** | Cross-sectional | Yes | Yes | Yes | Yes | Yes | Yes | Yes | Yes |
| **Barrimi et al** | Cross-sectional | Yes | Yes | Yes | Yes | Yes | Yes | Unclear | Yes |
| **Barrimi et al** | Cross-sectional | Yes | Yes | Yes | Yes | No | No | Unclear | Yes |
| **Barrimi et al** | Cross-sectional | Yes | Yes | Unclear | Unclear | Yes | Yes | Yes | Yes |
| **Umar et al** | Cross-sectional | Yes | Yes | Yes | Yes | Yes | Yes | Unclear | Yes |
| **Jemal et al** | Cross-sectional | Yes | Yes | Unclear | Unclear | Yes | Yes | Unclear | Yes |
| **Ogundipe et al** | Cross-sectional | Yes | No | Unclear | Unclear | Yes | Yes | Yes | Yes |
| **Ogunnubi et al** | Cross-sectional | Yes | Yes | Yes | Yes | Yes | Yes | Yes | Yes |
| **Aloba et al** | Cross-sectional | Yes | Yes | Yes | Yes | Yes | Yes | Yes | Yes |
| **Esan et al** | Cross-sectional | Yes | Yes | Yes | Yes | Yes | Yes | Yes | Yes |
| **Esan et al** | Cross-sectional | Yes | Yes | Yes | Yes | No | No | Unclear | Yes |
| El Oumary et al | Cross-sectional | Yes | Yes | Yes | Yes | No | No | Yes | Yes |
| **Oneib et al** | Cross-sectional | Yes | Yes | Yes | Yes | No | No | Yes | Yes |
| **Chlihfane et al** | Cross-sectional | Yes | Yes | Yes | Yes | No | No | Yes | Yes |
| **Mensi et al** | Cross-sectional | Yes | Yes | Yes | Yes | Yes | Yes | Yes | Yes |
| Khouadja et al | Cross-sectional | Yes | Yes | Yes | Yes | Yes | Yes | Unclear | Yes |
| **El Jabiry et al** | Cross-sectional | Yes | Yes | Yes | Yes | No | No | Yes | Yes |
| **Erfan et al** | Cross-sectional | Yes | No | Yes | Yes | No | No | Yes | Yes |
| **Naidoo et al** | Cross-sectional | No | Yes | Yes | Yes | Yes | Yes | Yes | Yes |
| **Bouhlel et al** | Cross-sectional | Yes | Yes | Yes | Yes | Yes | Yes | Yes | Yes |
| **Seid et al** | Cross-sectional | Yes | Yes | Yes | Yes | Yes | Yes | Yes | Yes |
| **Oriji et al** | Cross-sectional | Yes | No | Yes | Yes | Yes | Yes | Yes | Yes |
| Abraham et al | Cross-sectional | Yes | Yes | Yes | Yes | Yes | Yes | Unclear | Yes |
| **Fanta et al** | Cross-sectional | Yes | Yes | Yes | Yes | Yes | Yes | Yes | Yes |
| Hassan et al | Cohort | Yes | Yes | Yes | Yes | NA | NA | Yes | Yes |
| Alemu et al | Cross-sectional | Yes | Yes | Unclear | Unclear | Yes | Yes | Unclear | Yes |
| **Thungana et al** | Cross-sectional | Yes | Yes | Yes | Yes | No | No | Yes | Yes |
| **Hussien et al** | Cross-sectional | Yes | Yes | Yes | Unclear | Yes | Yes | Yes | Yes |
| **Weret et al** | Cross-sectional | No | Yes | Yes | Yes | Yes | Yes | Unclear | Yes |
| **Abbes et al** | Cross-sectional | Yes | Yes | Yes | Yes | Yes | Yes | Yes | Yes |

**NA*: Not applicable**

| **Authors** | **Study design** | Were the groups comparable other than the presence of disease in cases or the absence of disease in controls? | Were cases and controls matched appropriately? | Were the same criteria used for identification of cases and controls? | Was exposure measured in a standard, valid and reliable way? | Was exposure measured in the same way for cases and controls? | Were confounding factors identified? | Were strategies to deal with confounding factors stated? | Were outcomes assessed in a standard, valid and reliable way for cases and controls? | Was the exposure period of interest long enough to be meaningful? | Was appropriate statistical analysis used? |
| --- | --- | --- | --- | --- | --- | --- | --- | --- | --- | --- | --- |
| Kachouchi and al | Case-control | Yes | Yes | Yes | Yes | Yes | No | No | Yes | Yes | Yes |
| Vilyte et al | Case-control | No | No | Yes | Yes | Yes | Yes | Yes | Yes | Yes | Yes |


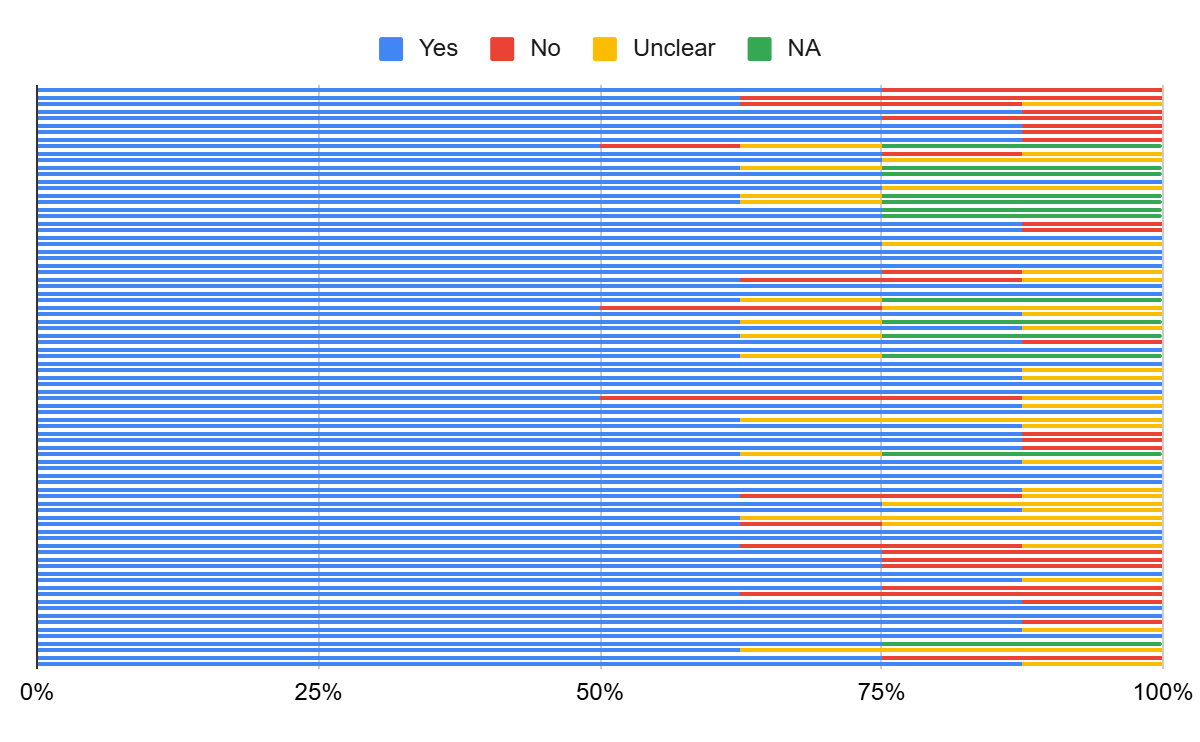


SM. Figure 1: Graphical representation of the quality assessment for cross-sectional and cohort studies

**SM.3: Sensitivity analysis by subgroup:**

**Lifetime suicide attempts :**


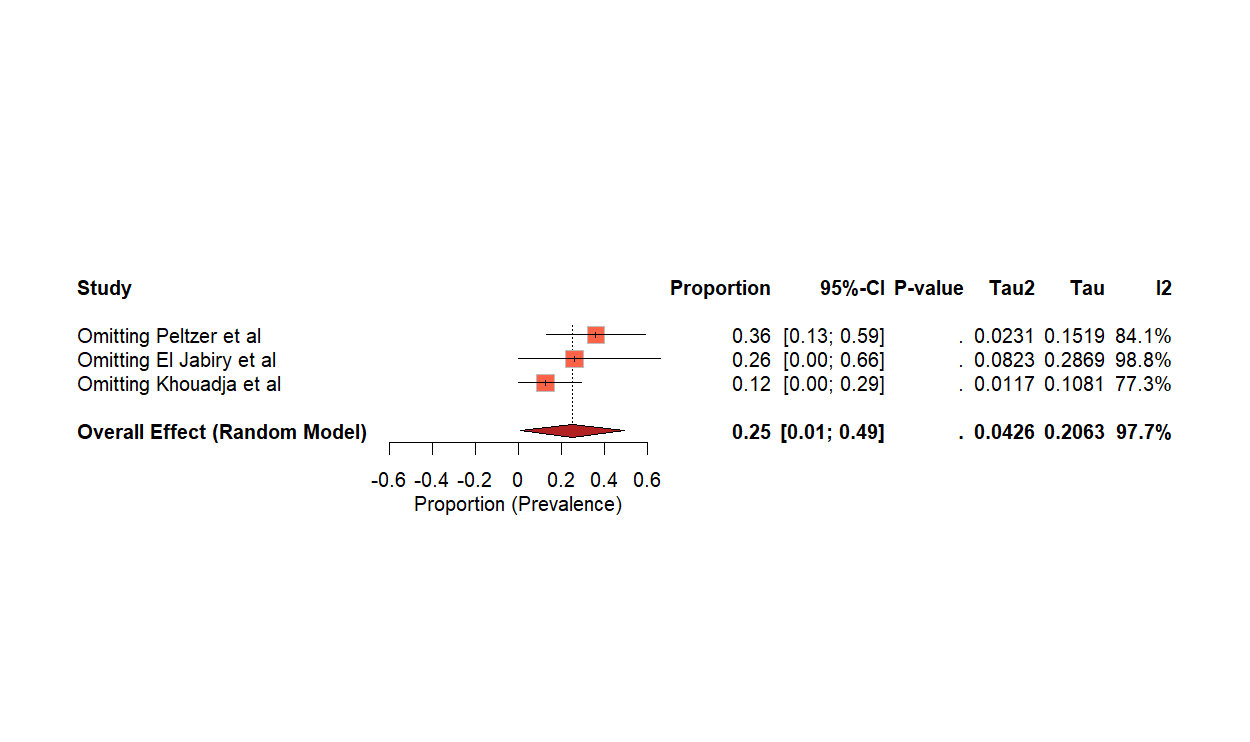


***Trauma and stressor related disorders***


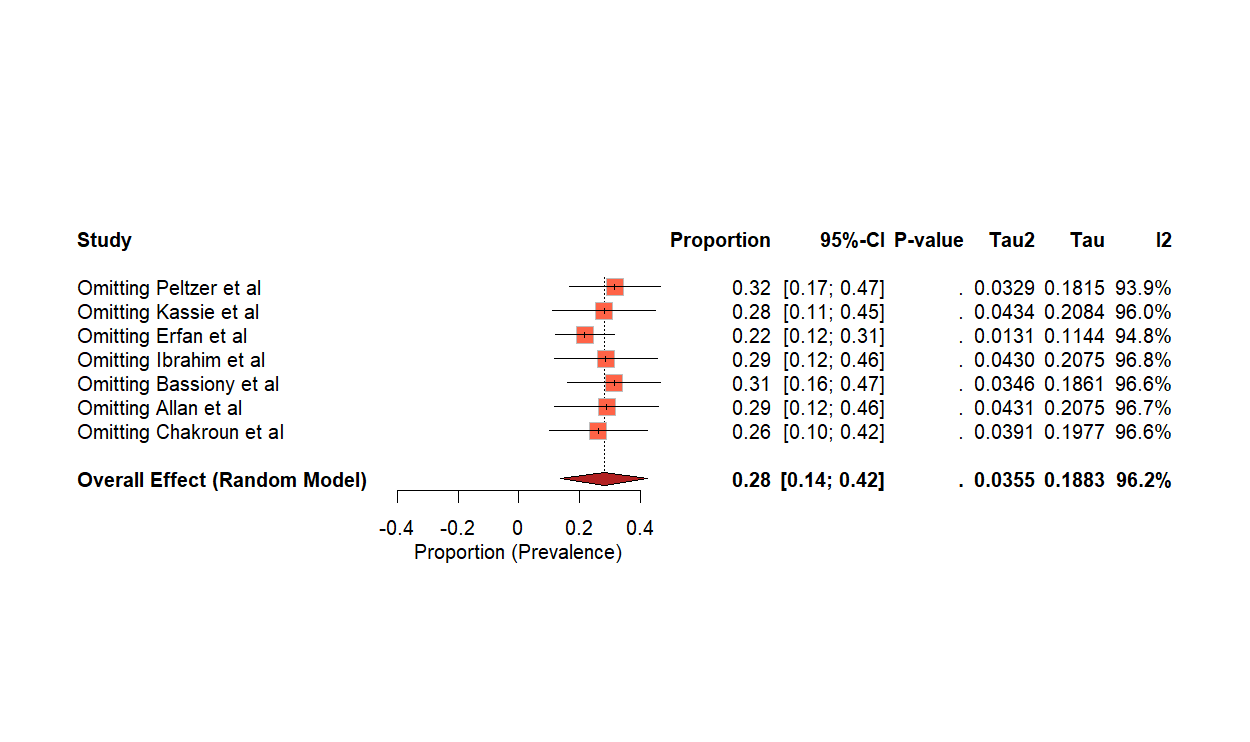


***Substance-related and addictive disorders****
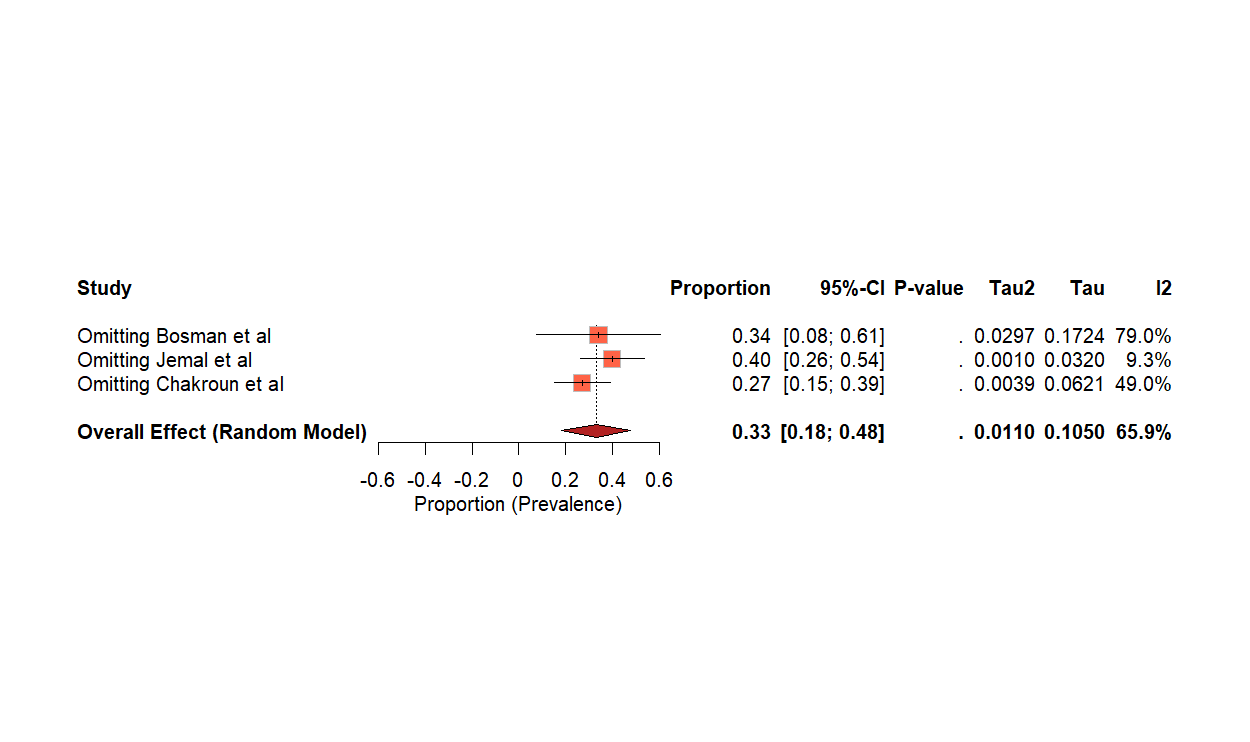
*

***Cluster B personality disorders***


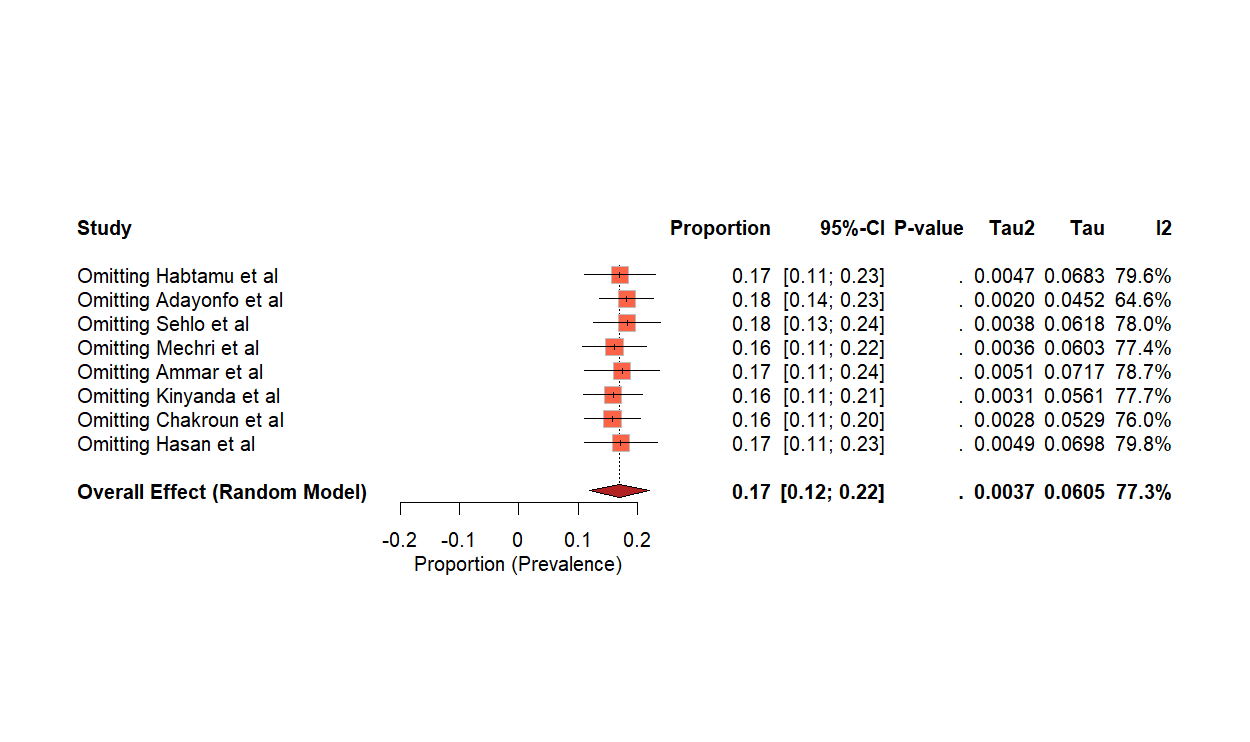


***Depressive disorders***


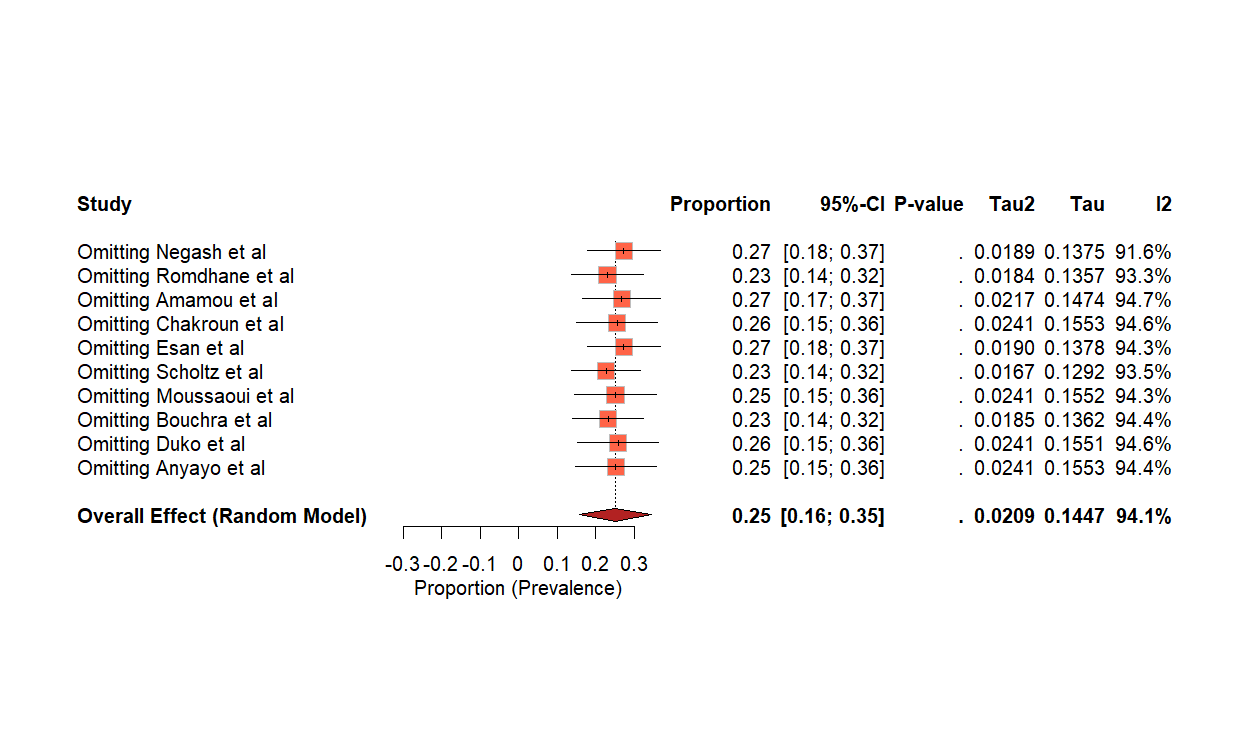

***Bipolar and related disorders***


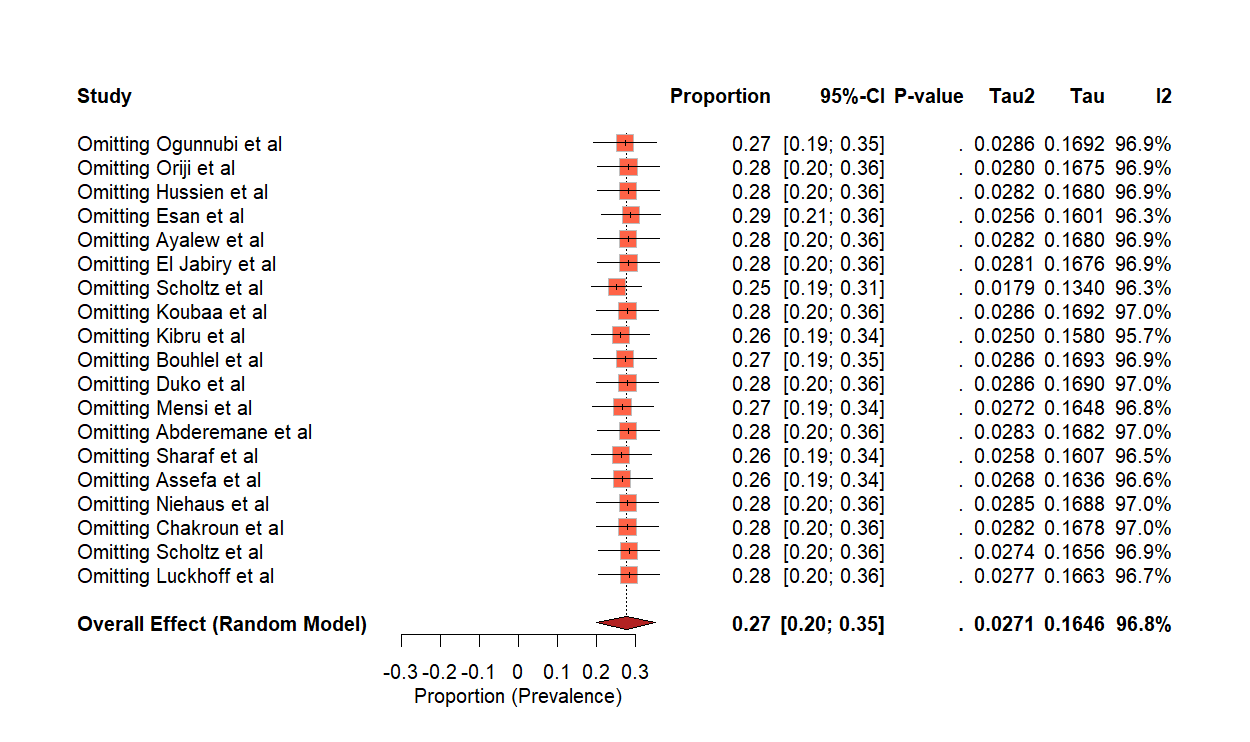


***Schizophrenia Spectrum and Other Psychotic Disorders***


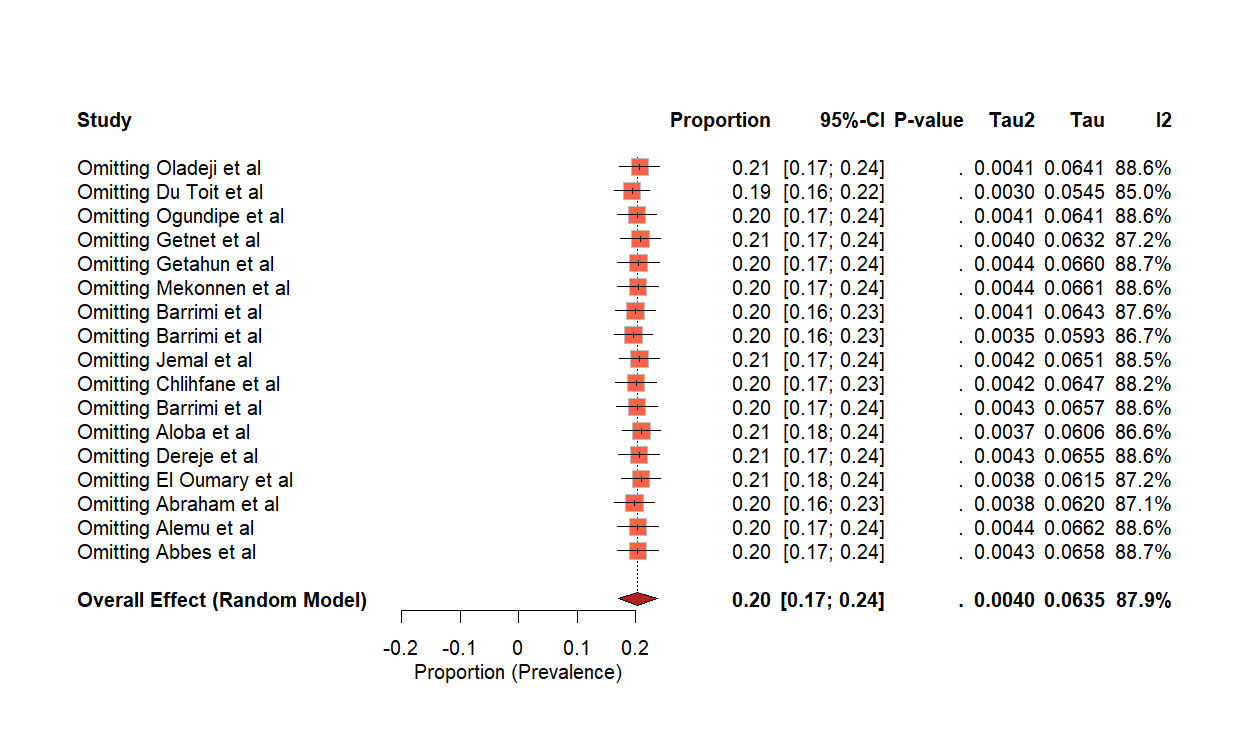


***Different psychiatric disorders***

**Recent suicide ideations:**


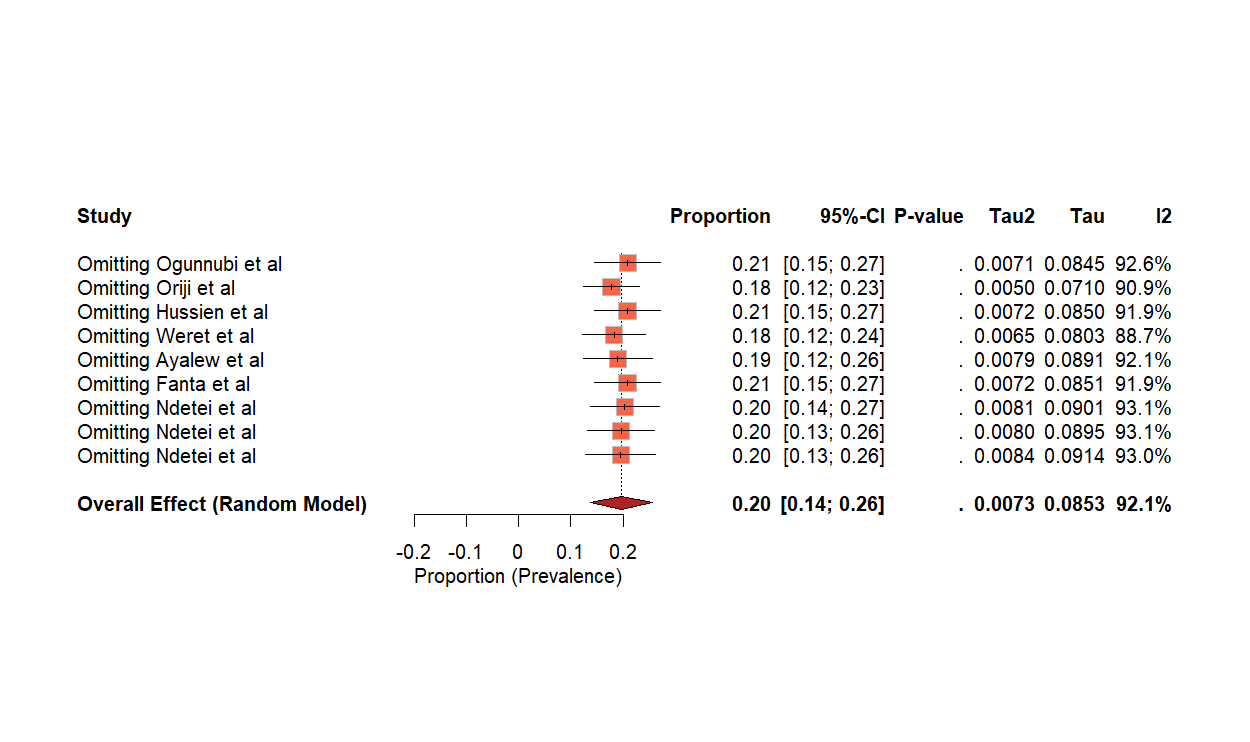


***Schizophrenia Spectrum and Other Psychotic Disorders***


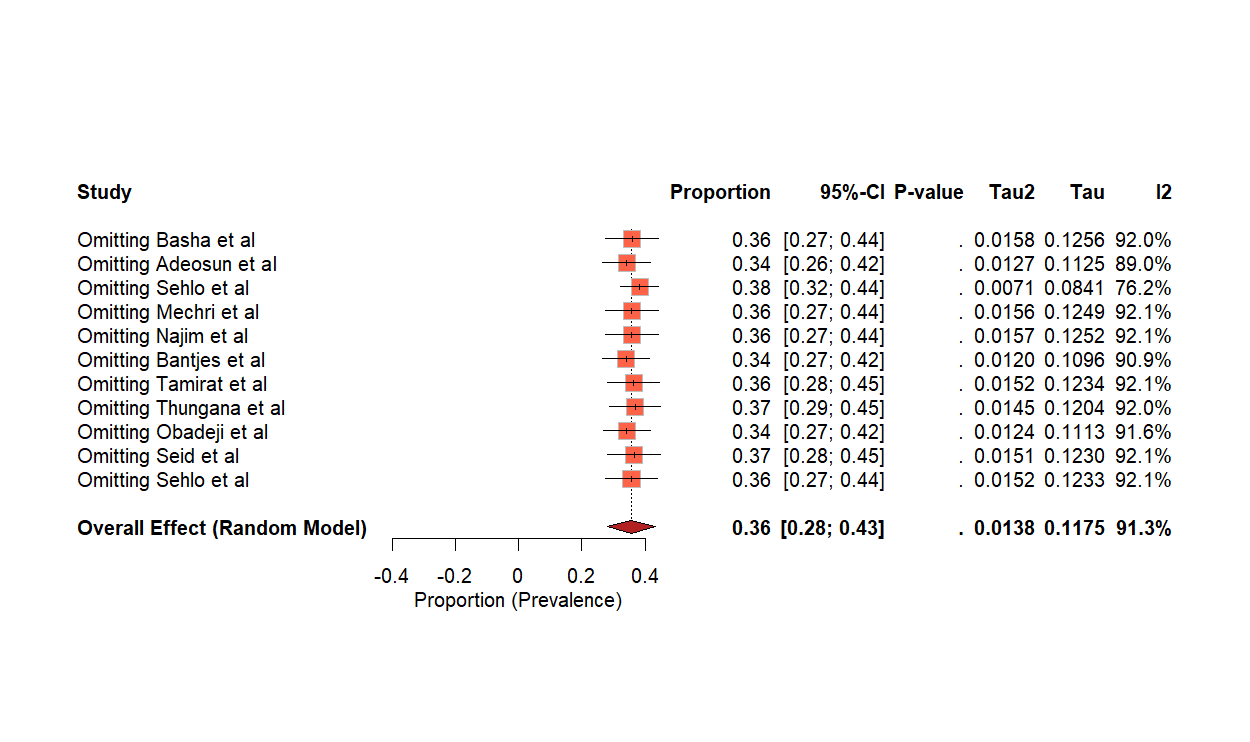


***Depressive disorders***


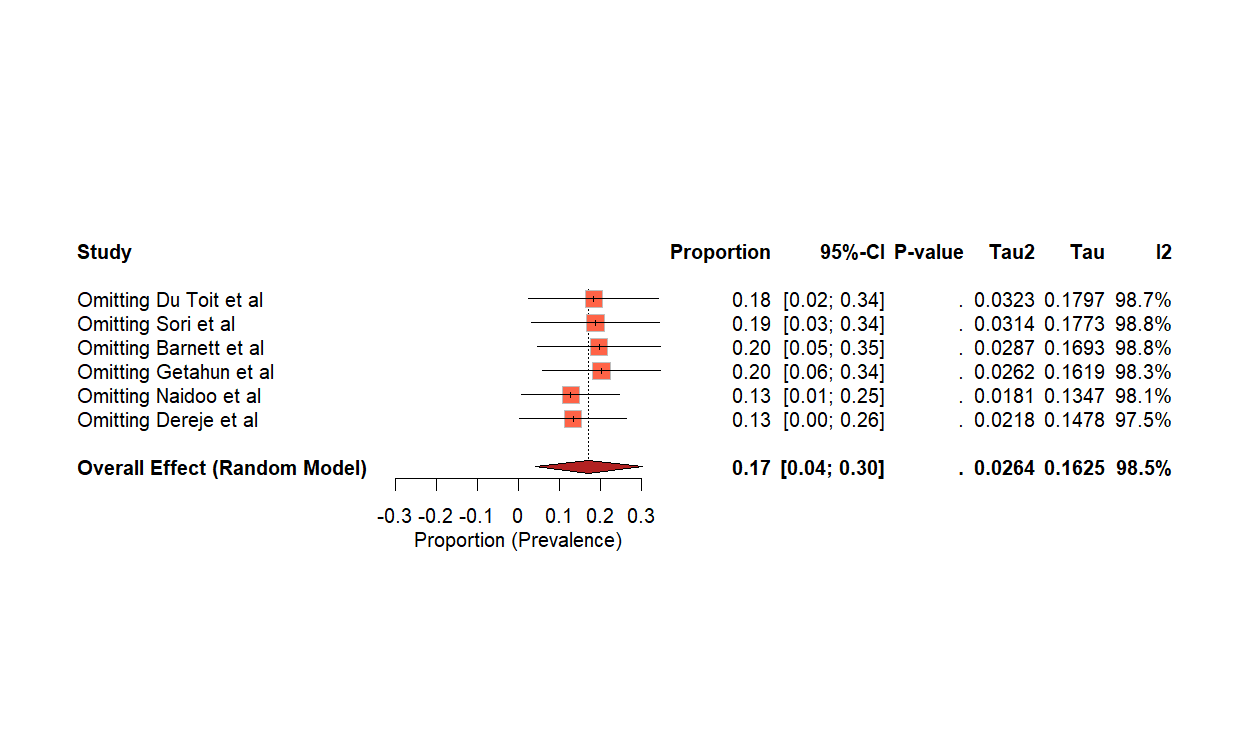


**Different psychiatric disorders**


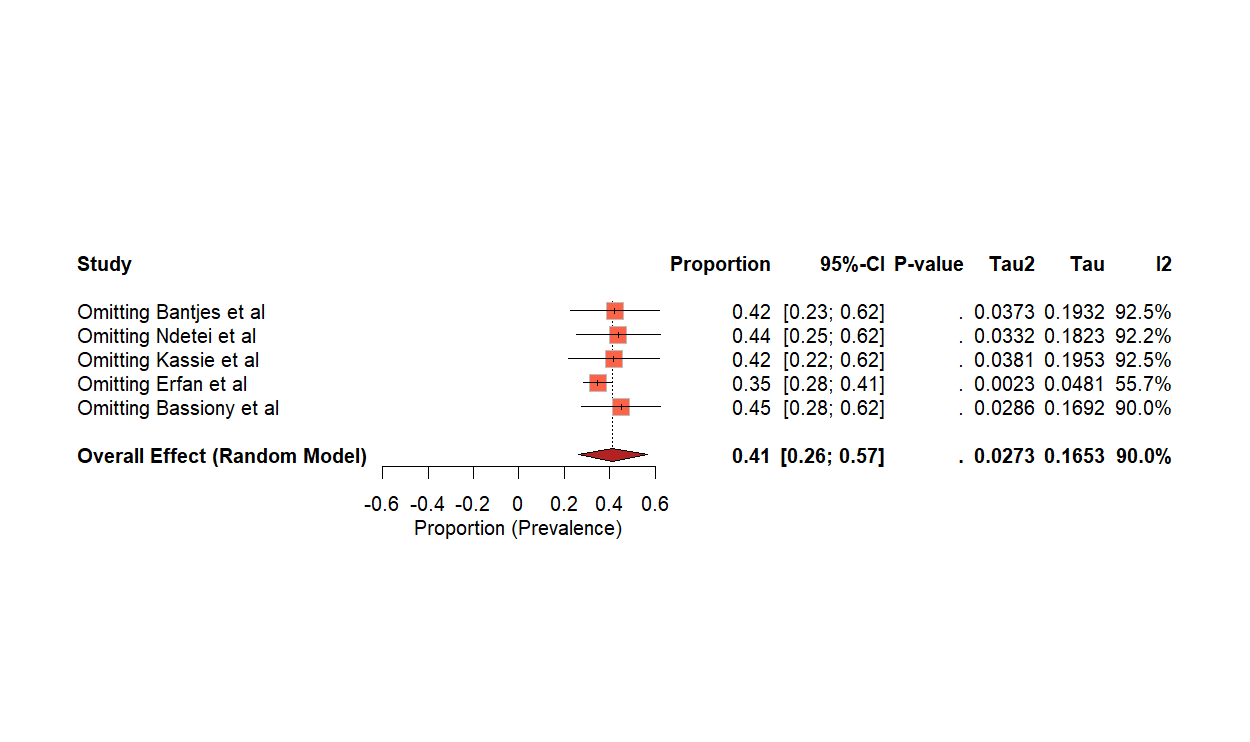


***Substance-related and addictive disorders***


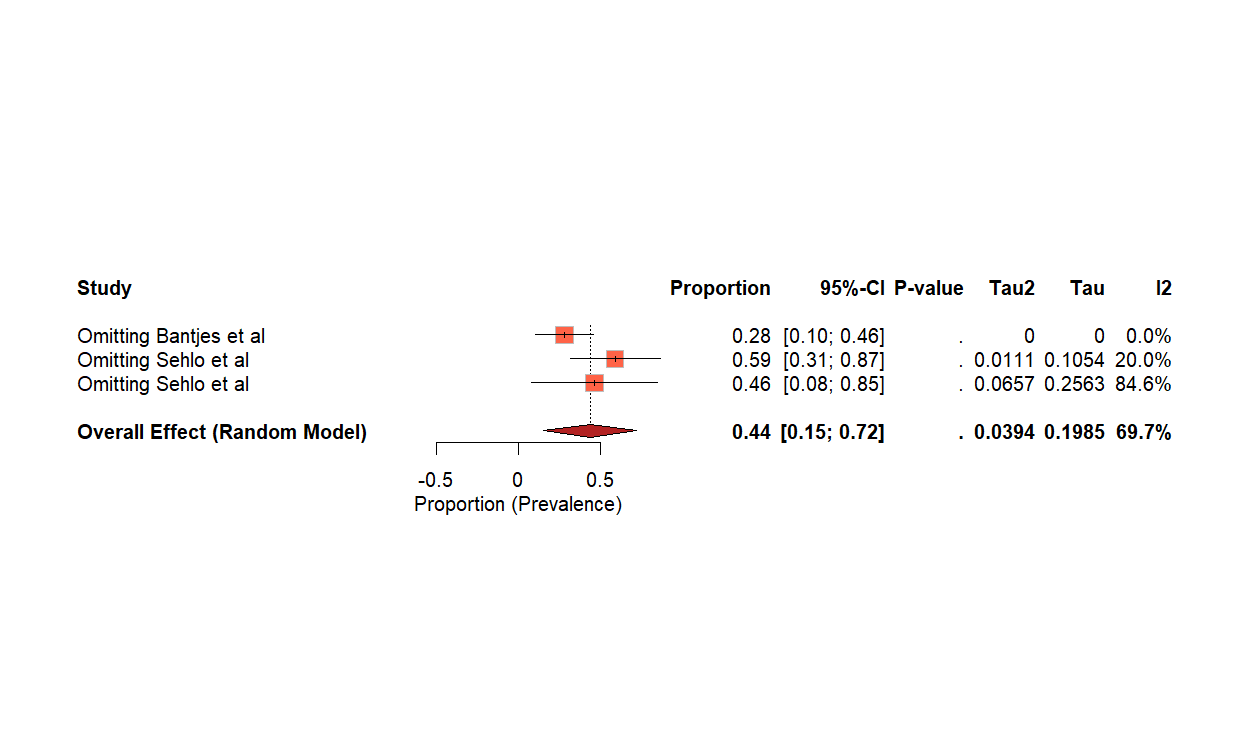


***Anxiety disorders***

**Lifetime suicide ideation :**


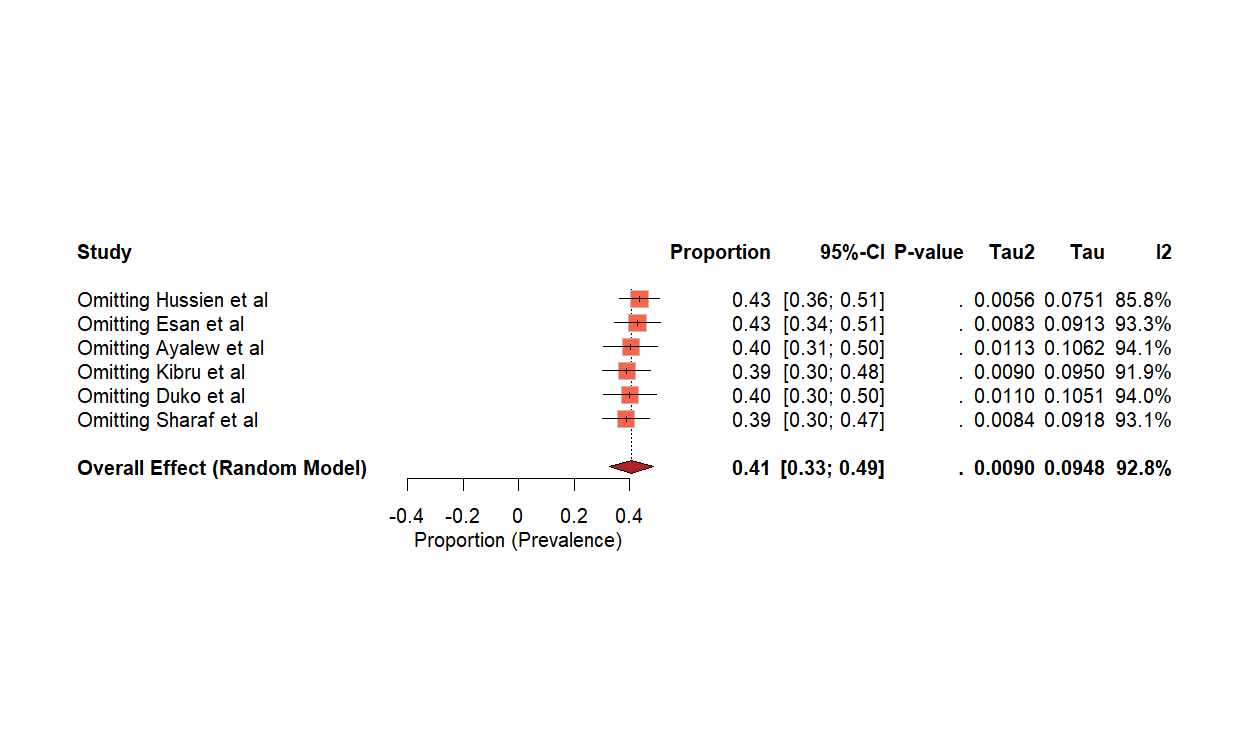


***Schizophrenia Spectrum and Other Psychotic Disorders***


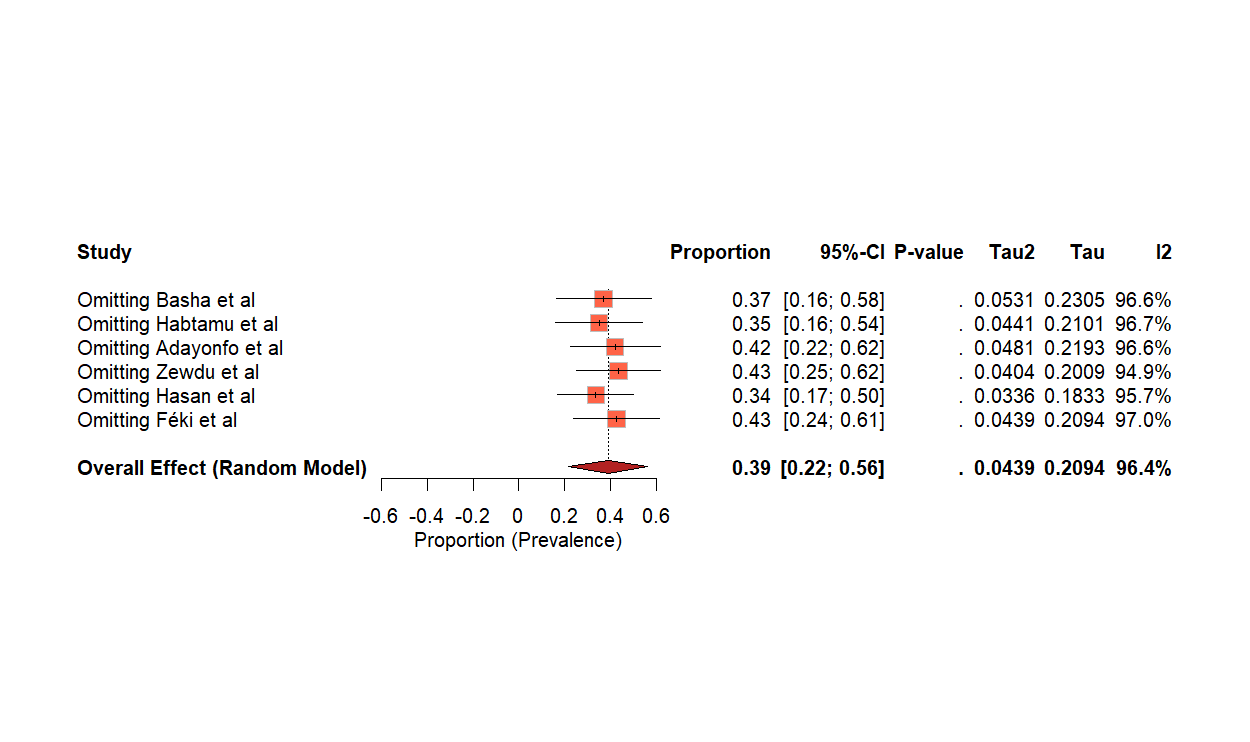


***Depressive disorders***


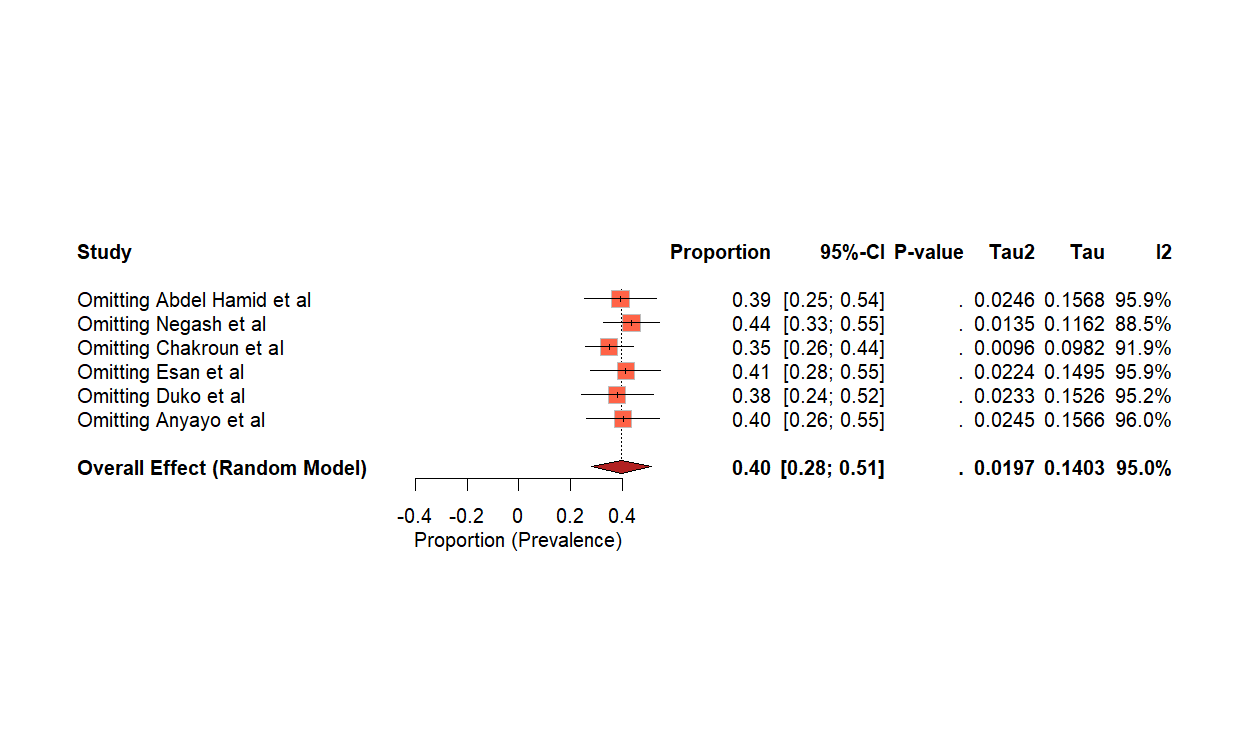


***Bipolar and related disorders***


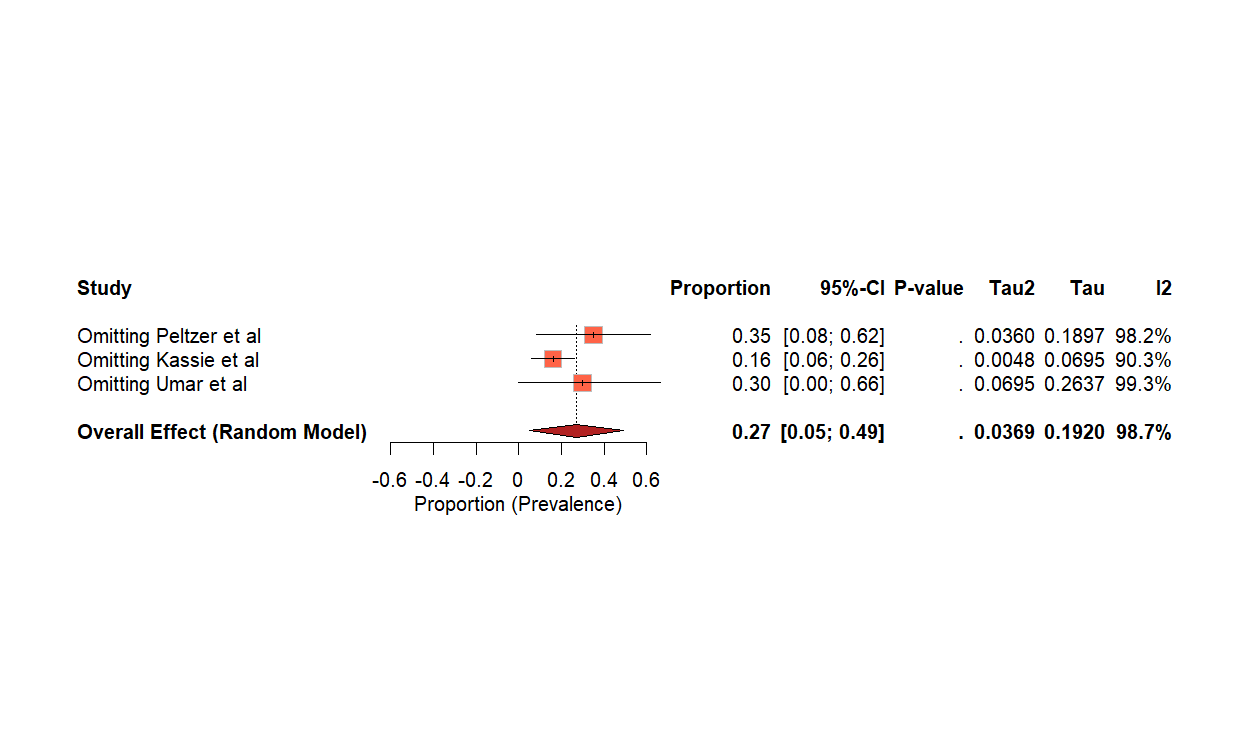


***Substance-related and addictive disorders***


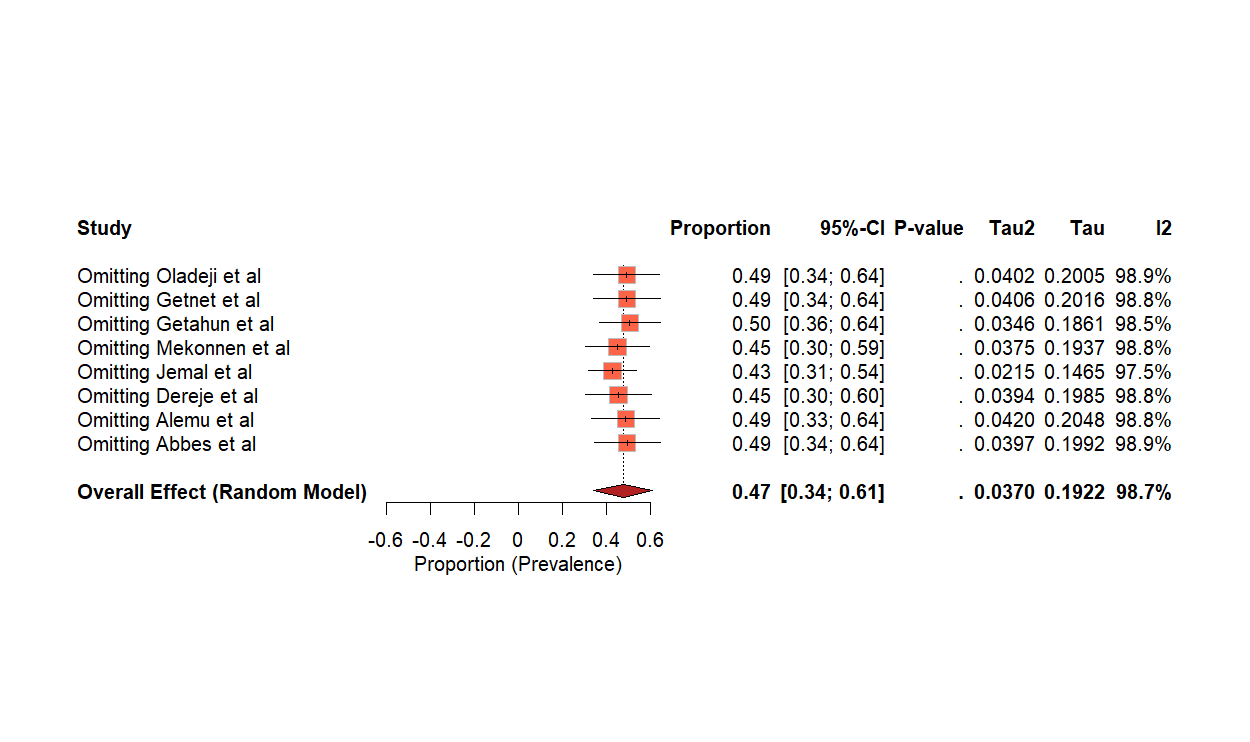


***Different psychiatric disorders***

**Recent suicide attempts:**


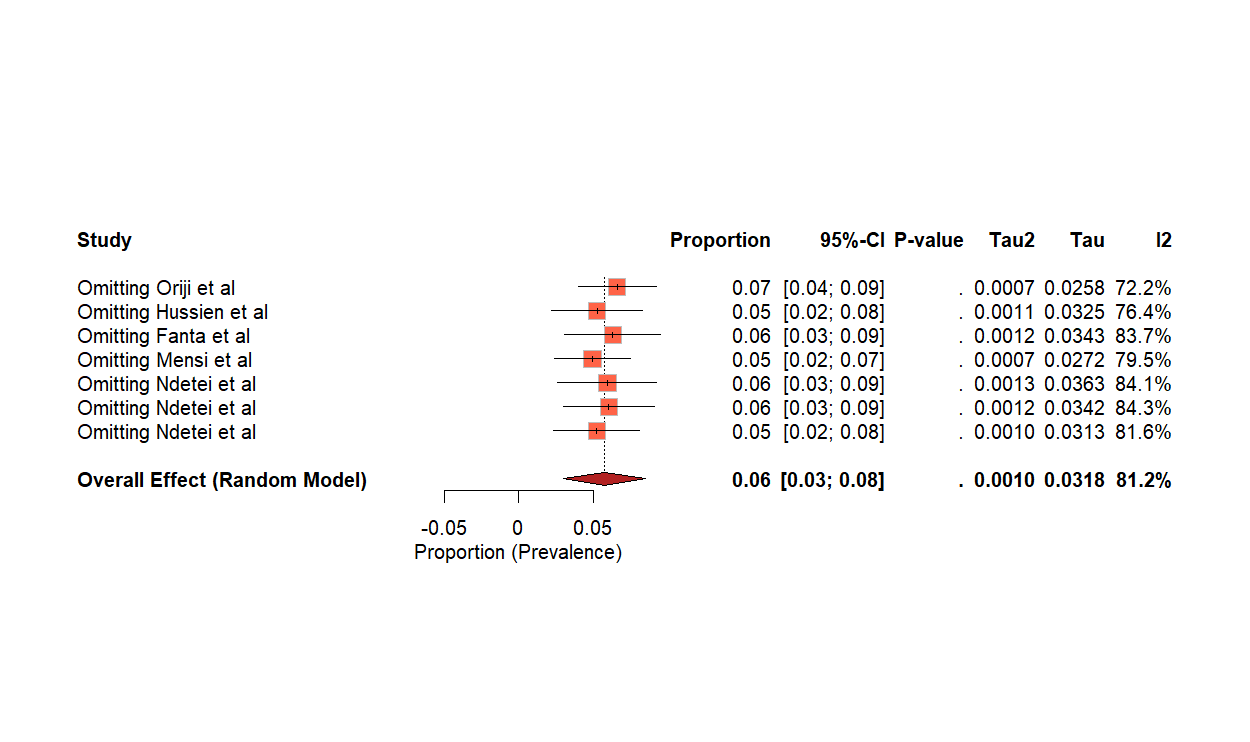


***Schizophrenia Spectrum and Other Psychotic Disorders***


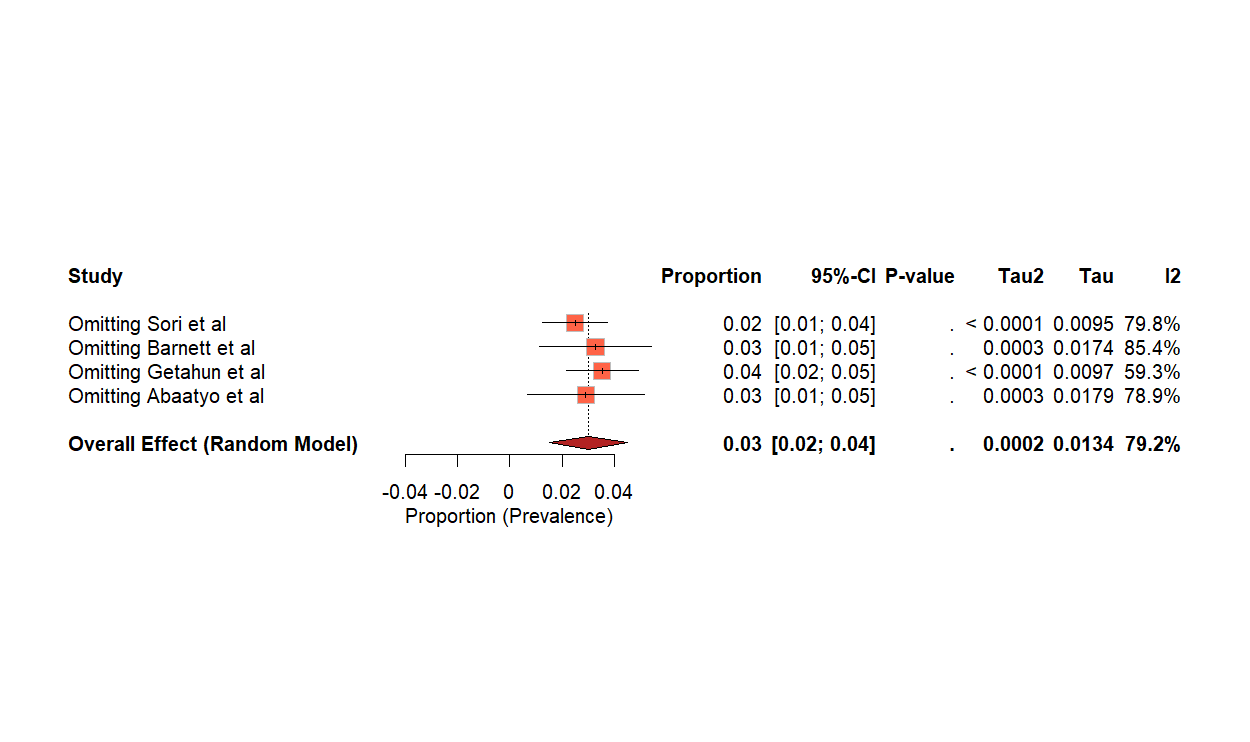


***Different psychiatric disorders***

**SM.4: Other subgroup Analysis (eg):**

**Lifetime suicide attempts:**


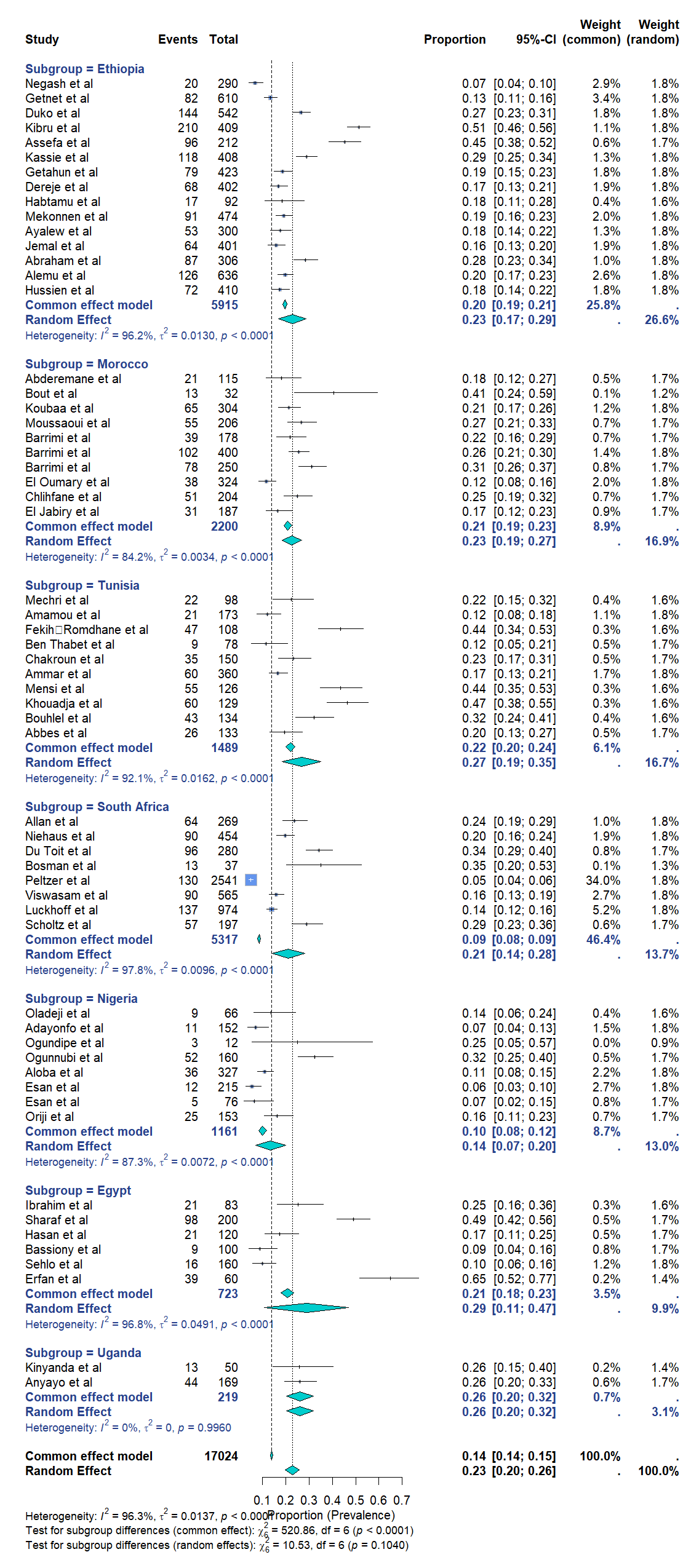

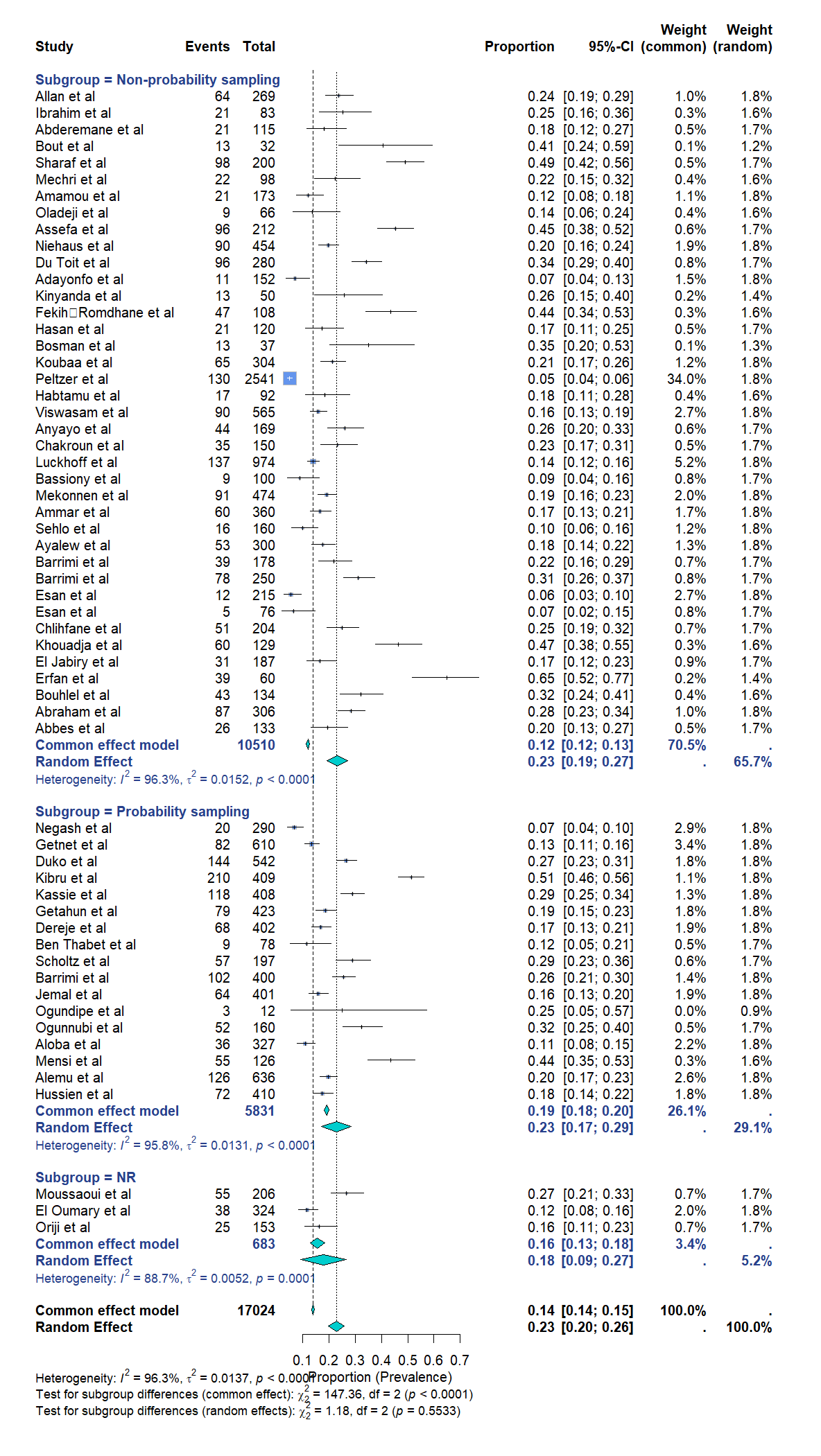


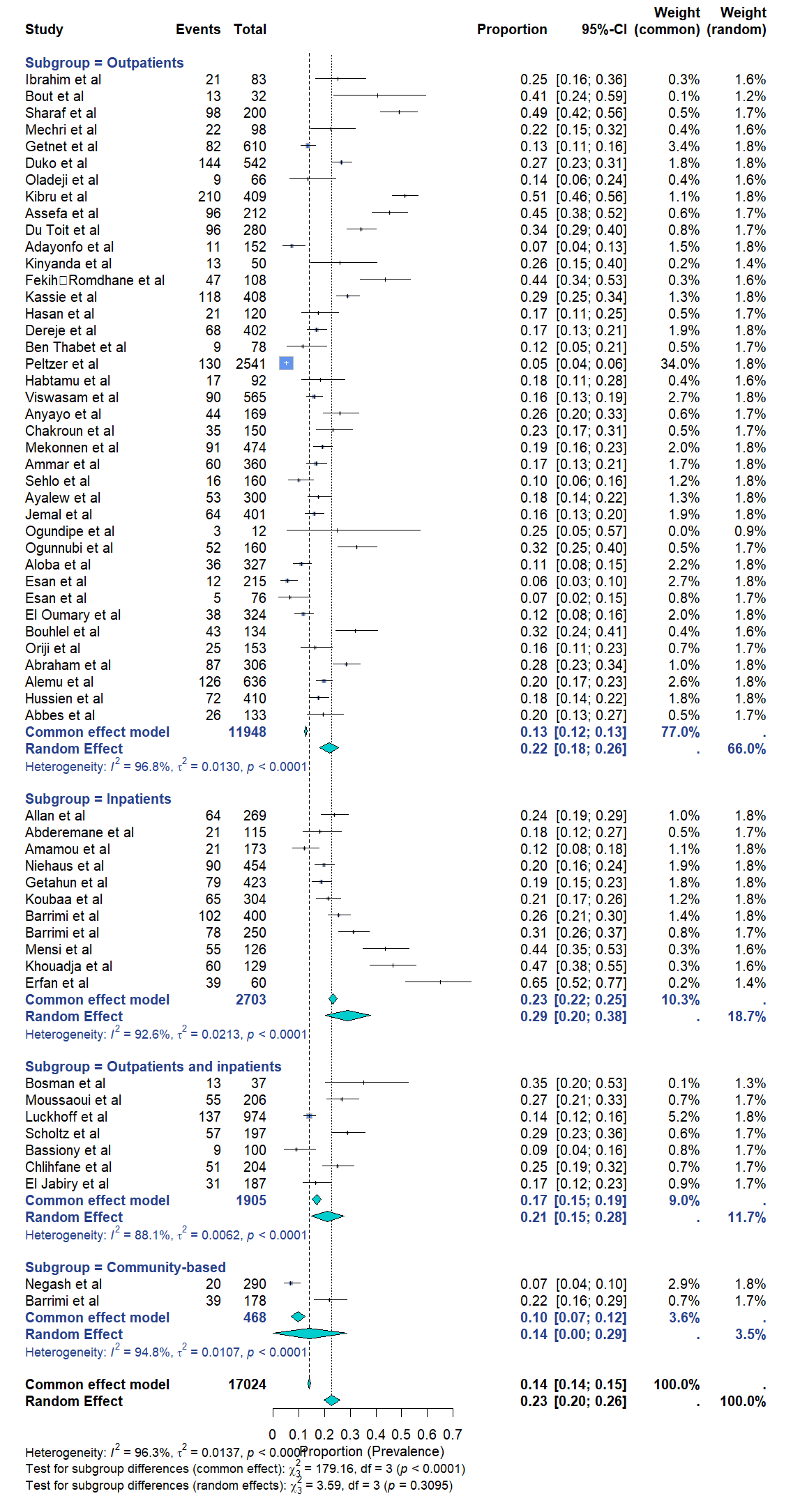

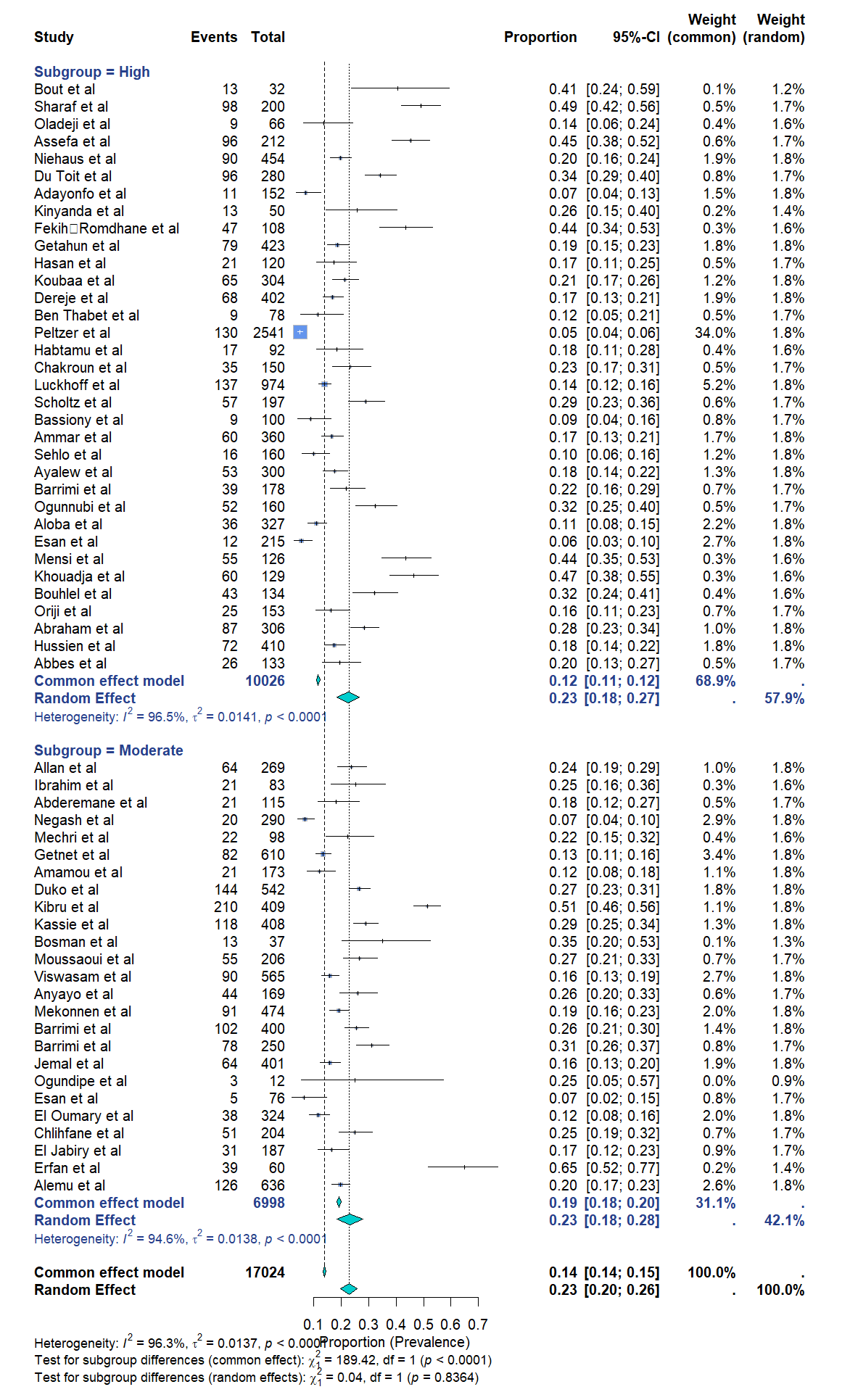


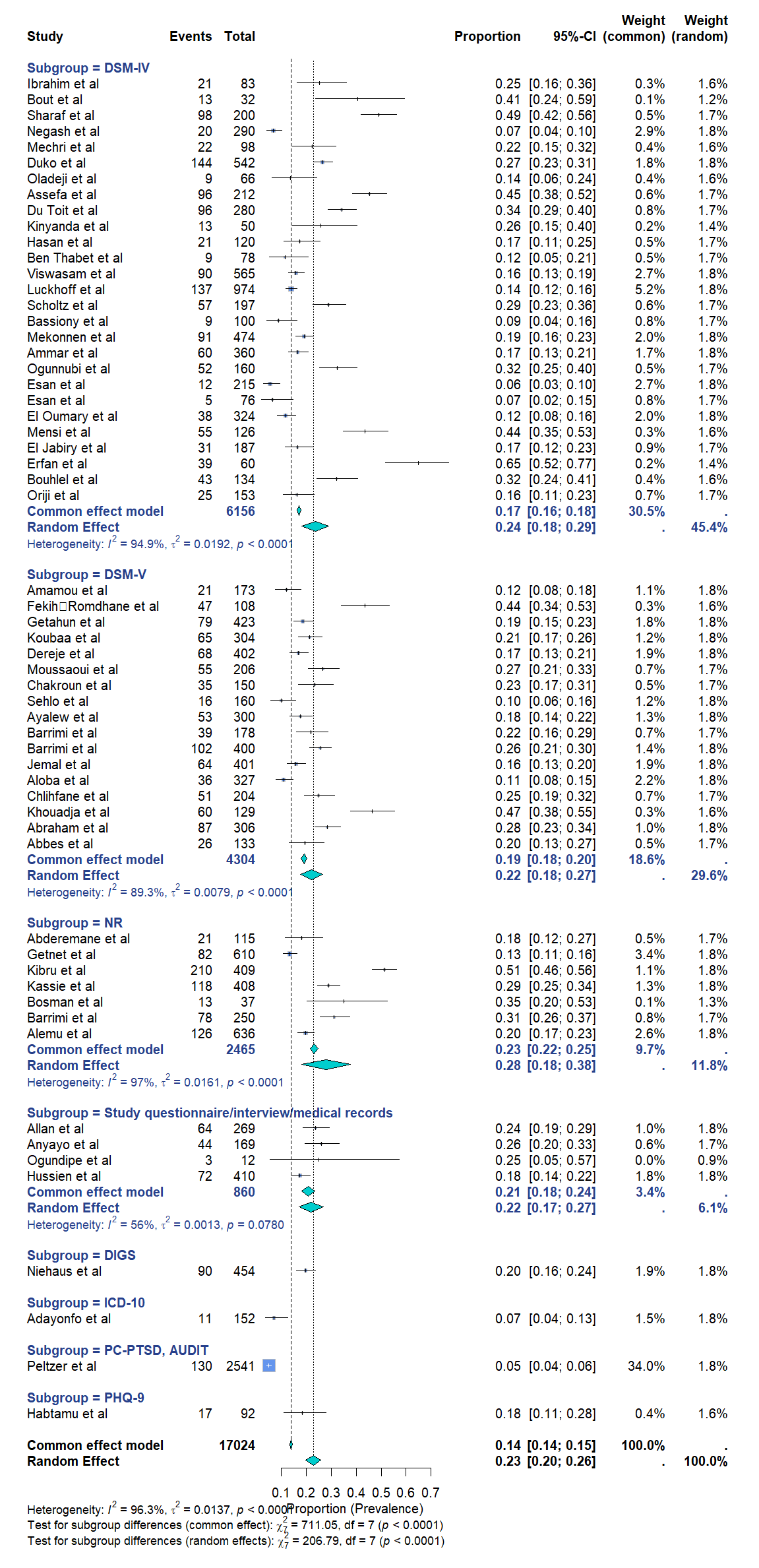

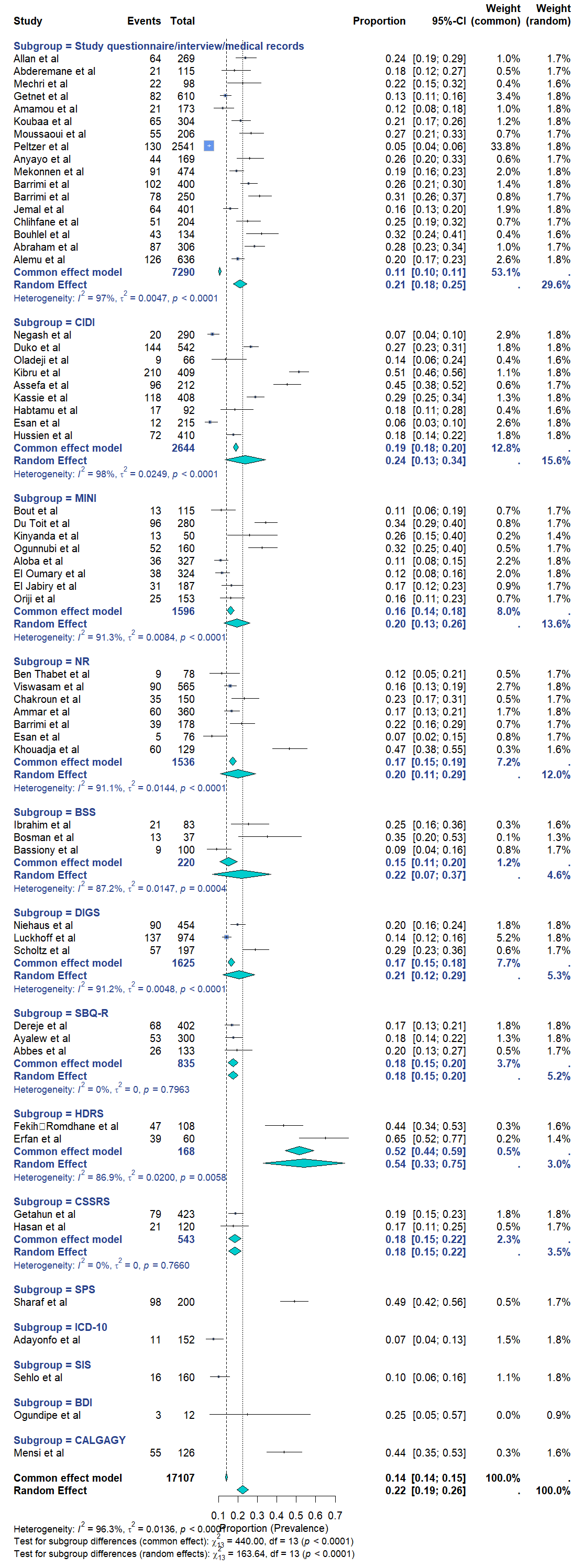


**SM.5 : Publication Bias assessed by Funnel Plots**


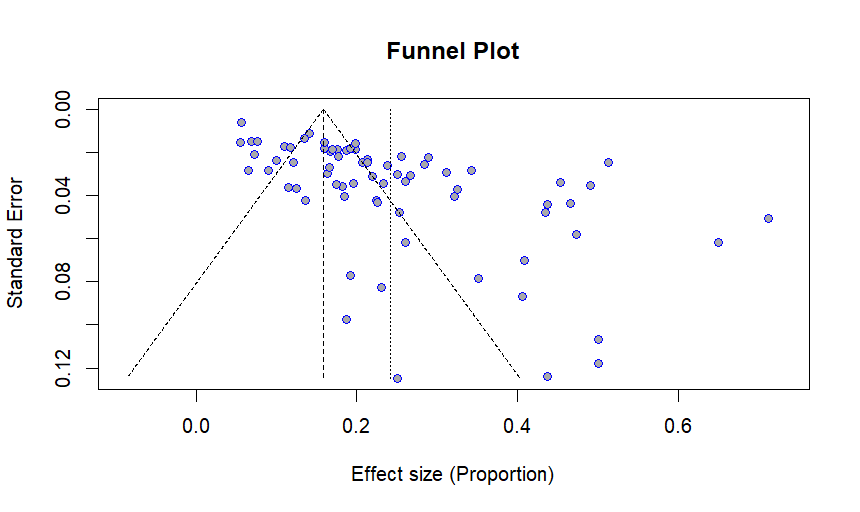


*Lifetime suicide attempts*


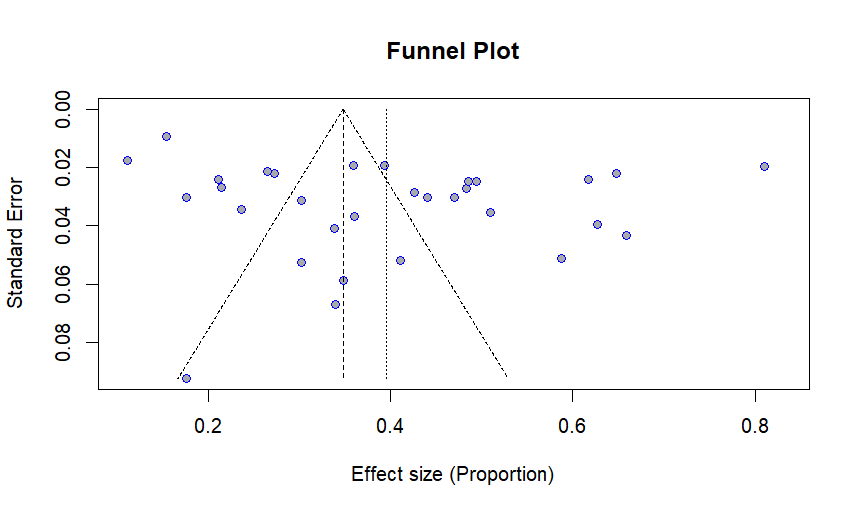


*Lifetime suicide ideation*


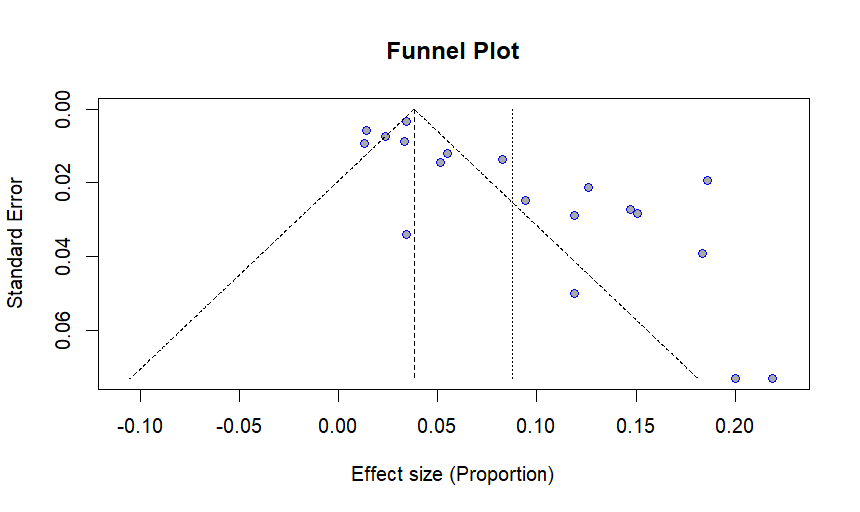


*Recent suicide attempts*


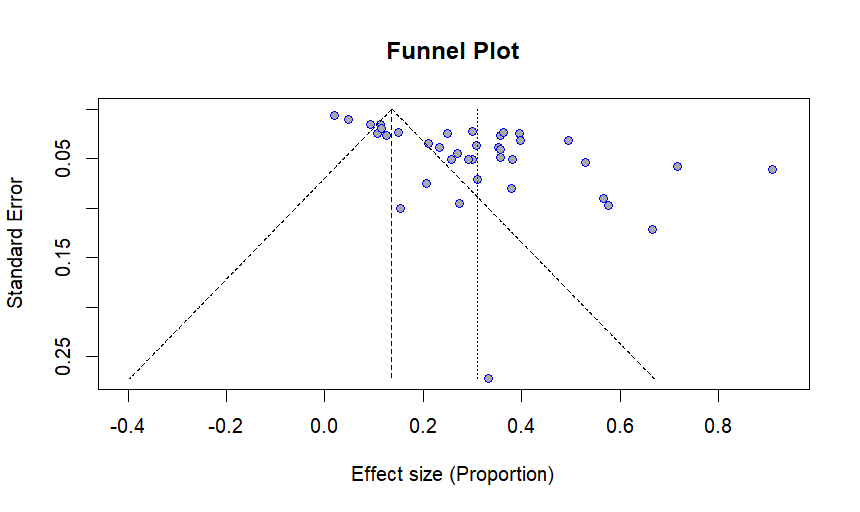


*Recent suicide ideation*
